# Recent progress of metal‐based nanomaterials with anti‐tumor biological effects for enhanced cancer therapy

**DOI:** 10.1002/EXP.20220001

**Published:** 2023-06-30

**Authors:** Huali Lei, Zifan Pei, Chenyu Jiang, Liang Cheng

**Affiliations:** ^1^ Institute of Functional Nano & Soft Materials (FUNSOM), Jiangsu Key Laboratory for Carbon‐Based Functional Materials and Devices Soochow University Suzhou China; ^2^ School of Optical and Electronic Information Suzhou City University Suzhou China; ^3^ Department of Chemistry North Carolina State University Raleigh North Carolina USA

**Keywords:** biological effects, biosafety, combined therapy, metal‐enhanced therapy, nanomaterials

## Abstract

Metal‐based nanomaterials have attracted broad attention recently due to their unique biological physical and chemical properties after entering tumor cells, namely biological effects. In particular, the abilities of Ca^2+^ to modulate T cell receptors activation, K^+^ to regulate stem cell differentiation, Mn^2+^ to activate the STING pathway, and Fe^2+/3+^ to induce tumor ferroptosis and enhance catalytic therapy, make the metal ions and metal‐based nanomaterials play crucial roles in the cancer treatments. Therefore, due to the superior advantages of metal‐based nanomaterials and the characteristics of the tumor microenvironment, we will summarize the recent progress of the anti‐tumor biological effects of metal‐based nanomaterials. Based on the different effects of metal‐based nanomaterials on tumor cells, this review mainly focuses on the following five aspects: (1) metal‐enhanced radiotherapy sensitization, (2) metal‐enhanced catalytic therapy, (3) metal‐enhanced ferroptosis, (4) metal‐enhanced pyroptosis, and (5) metal‐enhanced immunotherapy. At the same time, the shortcomings of the biological effects of metal‐based nanomaterials on tumor therapy are also discussed, and the future research directions have been prospected. The highlights of promising biosafety, potent efficacy on biological effects for tumor therapy, and the in‐depth various biological effects mechanism studies of metal‐based nanomaterials provide novel ideas for the future biological application of the nanomaterials.

## Graphical abstract

Metal‐based nanomaterials have attracted broad attention recently due to their unique biological physical and chemical properties after entering tumor cells, namely biological effects. Due to the superior advantages of metal‐based nanomaterials and the characteristics of the tumor microenvironment, we will summarize the recent progress of the anti‐tumor biological effects of metal‐based nanomaterials. Based on the different effects of metal‐based nanomaterials on tumor cells, this review mainly focuses on the following five aspects: (1) metal‐enhanced radiotherapy sensitization, (2) metal‐enhanced catalytic therapy, (3) metal‐enhanced ferroptosis, (4) metal‐enhanced pyroptosis, and (5) metal‐enhanced immunotherapy. At the same time, the shortcomings of the biological effects of metal‐based nanomaterials on tumor therapy are also discussed, and the future research directions have been prospected. The highlights of promising biosafety, potent efficacy on biological effects for tumor therapy, and the in‐depth various biological effects mechanism studies of metal‐based nanomaterials provide novel ideas for the future biological application of the nanomaterials.

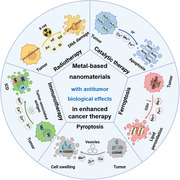

## 1 INTRODUCTION

Traditional tumor treatment methods including surgery, chemotherapy, and radiotherapy, although quite efficient and are the dominant therapies for cancer treatment, suffer from several severe limitations. For example, the surgery is difficult to completely remove the lesion and metastasis, while chemotherapy and radiotherapy would damage normal cells and have a wide variety side effects.^[^ ^1^ ^]^ Therefore, it is extremely urgent to develop novel tumor treatment methods. Current new tumor treatments, including radiotherapy sensitization to increase the sensitivity of tumor cells to x‐ray, photodynamic therapy, and photothermal therapy stimulated by laser, sonodynamic therapy stimulated by ultrasound (US), chemodynamic therapy (CDT) using Fenton catalysis, and so on, are inseparable from the involvement of metal‐based nanomaterials. Due to the comparable size of metal‐based nanomaterials to deoxyribonucleic acid (DNA), proteins, viruses, and biomolecules, there are special interactions between metal‐based nanoparticles and proteins in the matrix. Meanwhile, the metal‐based nanomaterials can also enter the blood circulation and penetrate into the tumor microenvironment (TME), and be further absorbed and transported by tumor cells.^[^ ^2^ ^]^ More importantly, the metal‐based nanomaterials with suitable size also have special enhanced permeability and retention (EPR) effect in the tumor sites, which further improves the utilization rate of nanomaterials and tumor treatment effect. Therefore, metal‐based nanomaterials with unique physical/chemical properties are crucial in the development of new cancer therapies.^[^ ^3^ ^]^

Due to the presence of membrane transporters, Na^+^/K^+^ pumps, carriers, and ion channels on the surface of cell membranes, there are various metal ion homeostasis in cells. Different from organic molecules, exogenous metals or metal ions are able to break intracellular ion homeostasis, or react with intracellular substances after entering cells to break intracellular redox homeostasis. For example, Fe^2+^ or Fe^3+^ will react with intracellular hydrogen peroxide (H_2_O_2_) or glutathione (GSH), respectively, after entering tumor cells to break the intracellular redox balance and cause cellular oxidative stress, thus inducing tumor cell death. Small amounts of Ca^2+^ are able to modulate T cell receptor activation by regulating the charge characteristics of lipids, while the overloading of Ca^2+^ will lead to immunogenic death of tumor cells.^[^ ^4^ ^]^ K^+^, Na^+^, and Ca^2+^ perform the capability of activating inflammasome, and K^+^ is also able to regulate stem cell differentiation.^[^ ^5^ ^]^ Fe^2+^/^3+^, Zn^2+^, Mn^2+^, and Cu^2+^ have the ability to affect the pathogen–host interface interaction. Mn^2+^ and Zn^2+^ could activate the cGSA‐STING pathway and enhance anti‐tumor immunotherapy.^[^ ^6^ ^]^ Taken together, different metal‐based nanomaterials are able to cause various biological effects. However, how to systematically develop a metal‐based nanomaterial that is able to effectively induce anti‐tumor biological effects, thereby enhancing tumor death while minimizing the normal tissue damage, remains a great challenge.

**SCHEME 1:**
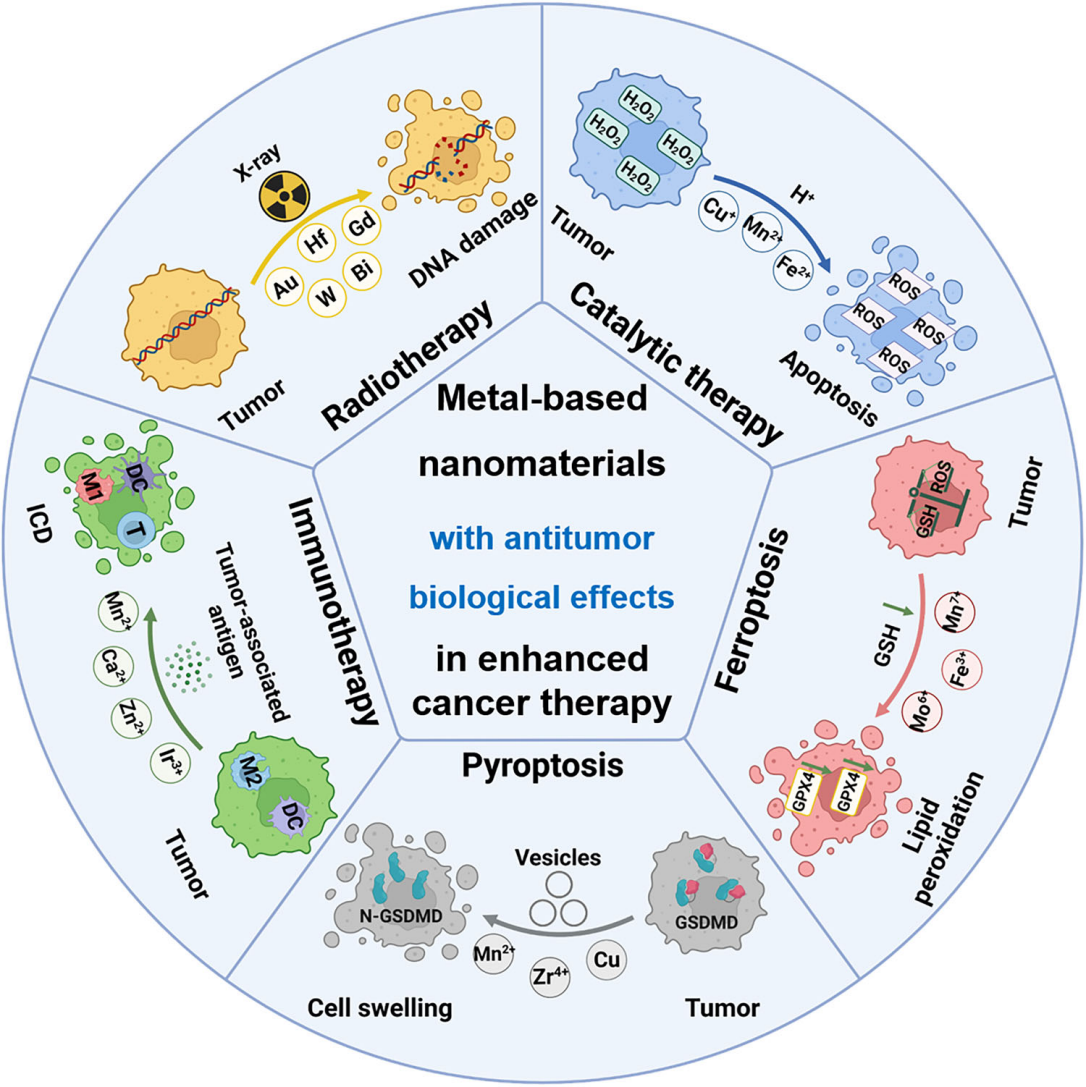
Metal‐based nanomaterials with anti‐tumor biological effects in cancer therapy for metal‐enhanced radiotherapy sensitization, metal‐enhanced catalytic therapy, metal‐enhanced ferroptosis, metal‐enhanced pyroptosis, and metal‐enhanced immunotherapy.

In recent years, based on the deep research in nanomaterials application on cancer treatment, more and more scientists paid attention to the biological effects of nanomaterials after entering the TME and tumor cells, providing new ideas for the design of nanomaterials for tumor therapy in the future. However, there are few reviews focusing on the biological effects of metal‐based nanomaterials in the synergistic tumor therapy. Therefore, based on the unique advantages attributed to the metal‐based nanomaterials, we summarized the anti‐tumor biological effects based on metal‐based nanomaterials, which were mainly divided into the following five directions: metal‐enhanced radiotherapy sensitization, metal‐enhanced catalytic therapy, metal‐enhanced ferroptosis, metal‐enhanced pyroptosis, and metal‐enhanced immunotherapy (Scheme 1). Moreover, the shortcomings of biological effects of metal‐based nanomaterials in tumor therapy and the future research directions had prospected, including the biosafety of metal‐based nanomaterials, the improving biological effects to enhance the effectiveness of tumor therapy, and the in‐depth study of various biological effects mechanism.

## 2 METAL‐BASED ENHANCED CANCER THERAPY

Similar to signal molecules or reactants, metal ions often participate in biological reactions and play an essential role in life activities. For example, copper can participate in the activities of more than a dozen enzymes in the body, which is able to organically combine tyrosine and tyrosinase with improving metabolic function and ensuring the generation and transfer of melanin. Iron is a vital part of hemoglobin, myoglobin, and iron‐containing oxidase, which plays an important role in the physiological activities. Zinc is an essential element for the normal growth and development of mammals. It is able to be used as an enzyme component, promote the development of sexual organs, promote appetite, participate in the synthesis of vitamin A and retinol‐binding protein, and participate in the body's immune function. In general, a metal ion performs a variety of functions in the matrix. Similarly, in the anti‐tumor treatment, a kind of metal is also able to play a variety of antineoplastic roles. For example, Fe ions not only have a Fenton reaction with H_2_O_2_ in the TME for tumor catalytic therapy, but also perform an ability to consume GSH in TME to break the redox balance of tumor cells and participate in metal‐enhanced ferroptosis, enlarge tumor cells, releases pyrosomes, and participates in metal‐enhanced pyroptosis.^[^ ^7^ ^]^ Therefore, based on the classification of different metal‐enhanced biological effects, we summarized the anti‐tumor biological effects enhanced by metal‐based nanomaterials, including metal‐enhanced radiotherapy sensitization, metal‐enhanced catalytic therapy, metal‐enhanced ferroptosis, metal‐enhanced pyroptosis, and metal‐enhanced immunotherapy. The representative metal‐based nanomaterials with anti‐tumor biological effects, among which Cu, Fe, Mn, Au, Gd, Mo, and Ir have played important roles, are listed in Table 1.

**TABLE 1:** Summary of biological effects of metal‐based nanomaterials in cancer treatment.

Types	Materials	Main metal element	In vitro	In vivo	Ref.
Radiotherapy sensitization	D‐iGSNPs	Au	GL261	GL261	[8]
	(M/H‐D) nanoradiosensitizer	Mo, Hf	SMMC‐7721/A549/THP‐1	SMMC‐7721‐fluc	[9]
	Zr‐MOF‐QU	Zn^2+^	HCC827/A549/MDA‐MB‐231	A549	[10]
	Biogenetic Au@MC38/ Au@4T1	Au	MC38/4T1/B16F10/ LLC	MC38/4T1	[11]
	{(Au^0^)_25_‐G5. NH_2_PS_20_}/siRNA polyplexes	Au^0^	A549	A549	[12]
	PVP‐PG	Bi/W	HeLa	HeLa	[13]
	HA@FeZOL	Fe^3+^	143B	143B	[14]
	Bi/Se‐Len NPs	Bi/Se	Hep3B/SK‐Hep1	H22	[15]
	HMTCP@PFP@O_2_	Ta/Cu	4T1	4T1	[16]
	Phy@PLGdH	Gd	CT26	CT26/4T1/Ranca	[17]
	c(RGDyC)‐AuNCs	Au	HeLa/MCF‐7	4T1	[18]
	HA@MOF/D‐Arg	Fe	K7M2	K7M2	[19]
	TaO_x_@Cat‐PEG	Ta	4T1	4T1	[20]
	PEG‐Bi_2_Se_3_@PFC@O_2_	Bi/Se	4T1	4T1	[21]
Catalytic therapy	Cu‐LDH/HMME@Lips,	Cu^2+^	4T1	4T1	[22]
	Co‐Fc@GOx	Fe^2+^	4T1	4T1	[23]
	Self‐supplying copper peroxide	Cu^2+^	4T1	4T1	[24]
	Copper(I) phosphide nanocrystals	Cu^+^	HeLa	U14	[25]
	Cu_2_‐xS‐PEG NDs	Cu^+^	4T1	4T1	[26]
	Single‐atom copper species	Cu	4T1	4T1	[27]
	DOX‐loaded CuCaP	Cu^+^	4T1	4T1	[28]
	Ferrous phosphide nanorods	Fe^2+^	HeLa	U14	[29]
	GOx@ZIF@MPN	Fe^2+^	4T1	4T1	[30]
	Amorphous iron nanoparticles	Fe	MCF‐7	4T1	[31]
	Tumor‐targeting iron sponge	Fe^2+^	4T1	4T1	[32]
	CS‐GOD@CM	Cu^+^	4T1	4T1	[33]
	HAS‐MnO_2_‐CuS	Cu^+^	Panc02	Panc02	[34]
	MS@MnO_2_ NPs	Mn^2+^	U87MG	U87MG	[35]
	CaO_2_−CuO_2_@HA NC	Cu^+^/Ca^2+^	4T1/B16F10/ CT26	4T1/B16F10/ CT26	[36]
	Fe‐VS_2_ NSs	Fe^2+^	4T1	4T1	[37]
	Fe‐TiO_2_ NDs	Fe^2+^	4T1	4T1	[38]
Ferroptosis	FeGd‐HN@Pt@LF/RGD2	Fe^2+^/ Fe^3+^	U‐87MG/ MCF‐7	U‐87MG	[39]
	PEGylated single‐atom Fe‐containing nanocatalysts	Fe	4T1	4T1	[40]
	CoMoO_4_‐phosphomolybdic acid nanosheet	Co^2+^/Mo^6+^	4T1	4T1	[41]
	Cro‐Fe@BSA	Fe^3+^	4T1	4T1	[42]
	Phototheranostic metal–phenolic networks	Fe^3+^	B16F10	B16F10	[43b]
	HCSVs	Fe^2+^/ Fe^3+^	TRAMP‐C1	TRAMP‐C1	[44]
	Ultrasmall FePt/siRNA	Fe^0^/Fe^2+^/ Fe^3+^	4T1	4T1	[43d]
	MnO*_x_* nanospikes	Mn	4T1	4T1	[45]
	VZnO	Zn	HCT116 /CT26	CT26	[46]
	Pa‐M/Ti‐NCs(m)	Fe^3+^/Fe^2+^	/	B16F10/4T1	[47]
	iRGD‐bcc‐USINPs	Fe	HepG2	HepG2	[48]
	Hybrid semiconducting nanozyme	Fe	4T1/HepG/SKOV3/MCF‐7/HeLa/PC12/231	4T1	[49]
	PtH@FeP	Fe^3+^	4T1	4T1	[43c]
	[Fe(III)salopheneCl]	Fe^3+^	HL60	/	[50]
	Fe‐Au DENP‐HQC/p53 polyplexes	Fe^3+^	PANC‐1	PANC‐1	[51]
	FSRSNs	Fe^3+^	4T1	4T1	[43a]
Pyroptosis	K_3_ZrF_7_:Yb/Er upconversion nanoparticles	K^+^/Zr^4+^	4T1	4T1	[52]
	PTAVs	Mn^2+^	4T1	4T1	[53]
	(M + H)@ZIF/HA	Zn	4T1	4T1	[54]
	COF‐909‐Cu	Cu	4T1	4T1	[55]
	VTPA	Mn^2+^/Fe^3+^	4T1	4T1	[56]
	MILH	Fe^2+^	INR1G9‐CCK2R/AGS‐CCK2R	/	[57]
	Lip‐MOF nanoparticles	Fe^3+^	HeLa	/	[58]
	CA‐Re	Re	MDA‐MB‐231	4T1	[59]
Immunotherapy	CMP_CDA_	Mn^2+^	/	CT26/B16F10/syngeneic squamous cell carcinoma	[60]
	Mn‐cGAMP NVs	Mn^2+^	/	B16F10	[61]
	^PEG^CaCUR	Ca^2+^	4T1	4T1	[62]
	PL/APMP‐DOX	Mn^2+^	4T1	4T1	[63]
	TiO_2_@CaP	Ca^2+^	4T1/A549	4T1	[64 ^]^
	NaGdF_4_:Nd@NaLuF_4_@PEG‐polyphenol/Mn MPN	Mn^2+^	4T1	4T1	[65]
	Iridium (III) complex	Ir	A549/A549R/LLC/MDA‐MB‐231/CT‐26	LLC	[66]
	FeOOH@STA/Cu‐LDH	Fe^2+^	4T1	4T1	[67]
	Au‐DOPC	Au	4T1	4T1	[68]
	DDMON‐IONP‐CUR	Ca^2+^	4T1	4T1	[69]
	Cu‐NCPs	Cu**^+^**/Cu^2^ **^+^**	CT26/4T1/HeLa	CT26/4T1	[70]
	CS‐I/J@CM	Cu/Se	GL261	GL261	[71]
	PSiNPs@Au	Au	4T1	4T1	[72]
	ZrO_2_‐x@PEG/cRGD	Zr	4T1	4T1	[73]
	UIONPs	Fe^3+^/Fe^2+^	4T1/B16‐OVA	4T1/B16‐OVA	[74]
	ZnPI‐PEGDOX	Zn^2+^	CT26/4T1	CT26	[75]

### 2.1 Metal‐enhanced radiotherapy sensitization

Radiotherapy is a prevalent anticancer therapy applied in clinical tumor treatment which employs α, β, γ and x rays to inactivate tumor cells and relieve the uncomfortable symptoms caused by the tumor compression. Conventional radiotherapy causes cell necrosis or apoptosis by generating cytotoxic hydroxyl radical (·OH) in tumor cells. However, its killing effect is not only on cancer cells, but also on normal cells near tumor tissues.^[^ ^76^ ^]^ At the same time, this therapy also limited by the hypoxia environment of many tumor tissues due to the insufficient blood supply. Under the hypoxia environment condition, the radiation would generate more hydroxide ion (OH^−^) without killing ability, rather than the biocidal ·OH, which greatly improves the tumor tolerance to radiation. In other words, hypoxic conditions would need higher doses of radiation for relatively good therapeutic effect and probably lead to more adverse effects on the normal tissues.^[^ ^77^ ^]^

To overcome this difficulty, radiosensitizers can be introduced into tumor cells to increase the sensitivity of the tumors to radiation. Therefore, the radiosensitizer nanomaterials with high‐Z elements, which are able to reduce the dose of x‐ray irradiation and improve the killing effect on the tumor, have attracted great attention. The ideal radiosensitizer nanomaterials must meet the following conditions: (1) Possess strong sensitization effect on tumor cells by radiotherapy. (2) Non‐ or low toxic and have no obvious sensitization effect to the normal cells. (3) Soluble and stable in the aqueous system. (4) Effectively penetrate into the tumor site. (5) Long biological half‐life to ensure reaching the tumor site.

**FIGURE 1:**
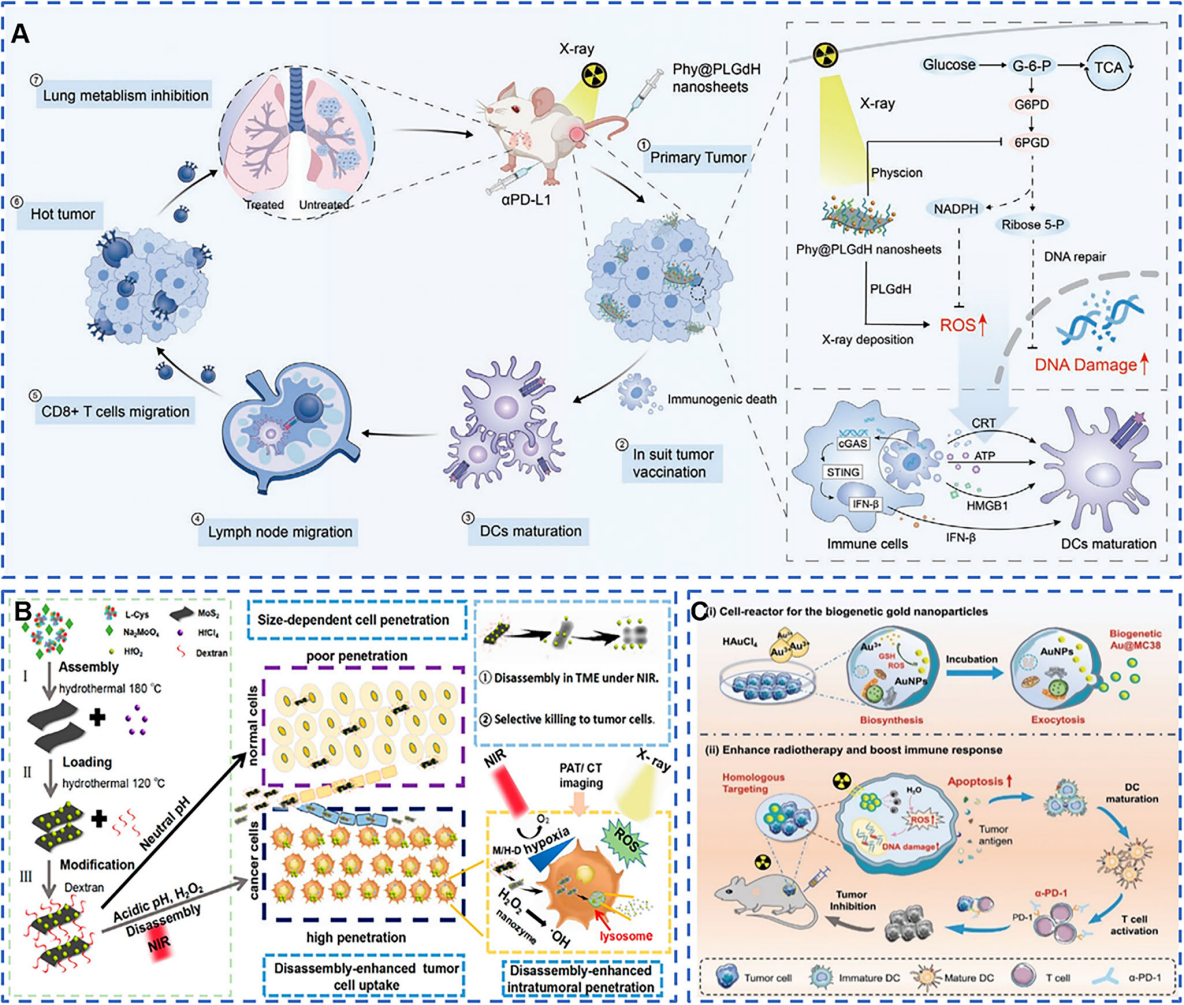
Metal‐based nanomaterials with radiotherapy‐sensitizing biological effects for cancer therapy. (A) Layered gadolinium hydroxide (Phy@PLGdH), which containing high‐Z element Gd, performed excellent x‐ray deposition ability, enhanced radiotherapy‐sensitizing biological effects, and was decomposed into small nanosheets or ligand complexes to reduce the toxic side effects on the kidney. Reproduced with permission.^[^ ^17^ ^]^ Copyright 2022, Wiley‐VCH GmbH. (B) Tumor microenvironment (TME)‐responsive biodegradable M/H‐D was internalized at the tumor site, effectively improving radiation efficiency, and was rapidly degraded in TME, greatly improving biosafety. Degraded Hf could also be further used for computed tomography imaging. Reproduced with permission.^[^ ^9^ ^]^ Copyright 2020, American Chemical Society. (C) Au@MC38 nanoparticles were prepared by using tumor cell membrane, which further enhanced the targeting of nanoparticles to tumor and enhanced the sensitization effect of radiotherapy. Reproduced with permission.^[^ ^11^ ^]^ Copyright 2021, Wiley‐VCH GmbH.

To address the problems that x‐ray is not deposited in tumor tissues sufficiently and only low levels of reactive oxygen species (ROS) can be generated to induce oxidative stress at recommended radiation doses, metal‐based radiosensitizers based on high‐Z elements are being widely developed for deposition of x‐ray for amplification of RT‐induced oxidative stress. For example, Au (Z = 79)‐based nanomaterials were widely used for radiotherapy sensitization because of their high inertness, good biocompatibility, and easy chemical modification. Rare‐earth nanoparticles with Z = 57–71, including Yb, Er, and Gd, among which Gd‐based nanoparticles had been widely used as magnetic resonance (MR) contrast agents in clinical settings with low toxicity and rapid clearance, and were potential radiosensitizers. Wang et al. synthesized the layered gadolinium hydroxides (LGdH) and modified them with DSPE‐PEG to obtain PLGdH with good water solubility (Figure 1A).^[^ ^17^ ^]^{Wang, 2022 #26} Meanwhile, in order to inhibit the pentose phosphate pathway (PPP), a branch of glycolysis that was overactive in most tumors, could provide abundant NADPH and ribose 5‐phosphate to maintain redox and nucleotide homeostasis, respectively, physcion (Phy, a PPP inhibitor) was further inserted between PLGdH layers to obtain Phy@PLGdH nanosheets. Phy@PLGdH nanosheets showed good stability in the physiological environment, however, Phy was gradually released to exert PPP inhibitory effects, as the pH decreased, while Gd^3+^ in PLGdH was not, which greatly reduced the metabolic toxicity of Gd^3+^. Due to the presence of Gd^3+^, Phy@PLGdH nanosheets exhibited excellent x‐ray deposition ability, disrupted the redox homeostasis of CT26 tumor cells, and showed superior in vitro radiosensitizing effects. More importantly, Phy@PLGdH nanosheets exhibited better sensitization to radiotherapy than that of the spherical Phy@PLGdH, suggesting that there was also a strong relationship between the biological effects of nanomaterials and the morphology. Simultaneously, in combination with PPP inhibitors Phy, Phy@PLGdH nanosheets showed excellent anti‐tumor effects in the CT26 tumor model. Finally, the Phy@PLGdH nanosheets would be decomposed into smaller nanosheets or ligand complexes and metabolized from urine, rather than in the form of Gd^3+^, further reducing the toxic side effects on the kidney.

To further improve the hypoxia‐related radiation therapy resistance due to the hypoxic TME, the introduction of oxygen generators along with the synthesis of nanomaterials containing high‐Z elements can further potentiate the radiation therapy sensitization effect. Song et al. prepared TaO*_x_* hollow nanospheres by the one‐step method at room temperature and added catalase during the synthesis process, so that the catalase was loaded inside the nanospheres, and then modified via layer by layer method to obtain stable TaO*_x_* @Cat nanospheres.^[^ ^20^ ^]^ TaO*_x_* shell maintained the activity of catalase and catalyze the production of O_2_ from highly expressed H_2_O_2_ only at the tumor site, improving the hypoxic tumor environment, while sufficient O_2_ was able to further potentiate the radiotherapy sensitizing effect of the TaO*_x_* shell. TaO*_x_* @Cat had demonstrated the ability to damage double‐strand DNA in tumor cell and inhibit tumor cell proliferation. More importantly, TaO*_x_* @Cat had the ability to perform a powerful effect of overcoming tumor hypoxia. In addition, increasing blood flow to the tumor site by the mild photothermal effect has become one of the approaches to improve tumor hypoxia and potentiate the sensitizing effect of radiotherapy. Song et al. also synthesized Bi_2_Se_3_ nanoparticles with hollow structure using the cation exchange method, the loaded perfluorocarbon (PFC) was employed as the oxygen reservoir in Bi_2_Se_3_ shell to obtain biocompatible Bi_2_Se_3_@PFC@O_2_.^[^ ^21^ ^]^ Bi_2_Se_3_ shell had strong absorption in near infrared and perform good photothermal properties, which could generate heat under NIR light irradiation, increase the blood flow, and overcome hypoxia at the tumor site. Bi_2_Se_3_@PFC@O_2_ had an ability to exhibit a good effect on the induction of DNA damage in tumor cells both in vitro and in vivo. To relieve the safety concern of metal‐based nanomaterials, food and drug administration (FDA) approved Hf‐based radiotherapy sensitizers were modified for cancer radiation therapy. Hafnium oxide nanoparticles have been approved in Europe for the treatment of locally advanced soft tissue sarcomas and have attracted great interest due to their high x‐ray coefficients, negligible toxicity, and chemical inertness. At the same time, TME‐responsive biodegradable metal‐based nanomaterials were also applied to tumor radiotherapy sensitization in order to accelerate the organism's metabolism. MoS_2_/HfO_2_ was obtained by using a two‐step hydrolysis method to anchor HfO_2_ on the surface of the synthesized MoS_2_ and modulating the amount of HfO_2_ anchoring by adjusting the amount of Hf^4+^ source (Figure 1B).^[^ ^9^ ^]^ Finally, the biodegradable dextrose (D) was modified to improve the water solubility and biosafety of MoS_2_/HfO_2_, resulting in MoS_2_/HfO_2_‐dextrose (M/H‐D). The photothermal property of MoS_2_ in M/H‐D promoted the internalization of HfO_2_ in the tumor resulting in higher yield of ROS, improved radiation efficiency and less damage to the normal tissues. M/H‐D showed excellent radiosensitizing effects in a variety of tumor cell lines, such as SMMC‐7721 and A549. Meanwhile, the acidic conditions (pH = 5.5–6.5) and high H_2_O_2_ concentration (100 μM) in the TME were able to accelerate the degradation of MoS_2_, and the x‐ray further improved the degradation rate of MoS_2_, therefore, MoS_2_ would be rapidly excreted after the tumor‐killing effect, which improved the biosafety of the nanomaterials. The degraded Hf also showed excellent computed tomography (CT) contrast ability, which further provided impact support for the tumor radiotherapy.

Since most of the metal elements with radiotherapy sensitization are heavy elements with high atomic numbers, although metal‐based nanomaterials show good EPR effect in tumor sites, they still perform a certain level of toxicity after being i.v. injected. In order to improve the biosafety of nanomaterials, biosynthetic nanomaterials, including various microorganisms, such as bacteria, fungi, and yeast, are gradually recognized. However, substances such as lipopolysaccharides expressed on the surface of these microbial membranes may cause inflammatory responses upon entry into the body. Meanwhile, most cells can secrete extracellular vesicles (EVs) either naturally or in response to external stimuli for transporting various endogenous and functional substances, creating new possibilities for the application of biosynthetic nanomaterials.^[^ ^78^ ^]^ Therefore, for the purpose of improving the homology of the biosynthetic materials, reducing the immune response, and enhancing the homologous tumor targeting in tumor therapy, metal‐based nanomaterials prepared or modified with different tumor cells were widely used in anti‐tumor therapy. Qin et al. prepared tumor cell‐derived Au@MC38 nanoparticles using MC38 cells as the parental cell and HAuCl_4_ as the Au source (Figure 1C).^[^ ^11^ ^]^ In this process, GSH in tumor cells was found to function as a metal‐binding protein for metal metabolism, while ROS participated in nanoscale biomineralization by providing additional dots to metal ions, thus jointly inducing the preparation of biosynthetic Au nanoparticles. Meanwhile, to verify the generality of biosynthesizing Au NPs, walled cells (LLC cells and 4T1 cells), suspension cells (H22 cells and HL‐60 cells), and immune cells (Raw264.7 cells and DC2.4 cells) were utilized to prepare Au NPs and showed commensurate biosynthetic ability. Due to the presence of Au, Au@MC38 NPs exhibited a good radiosensitizing effect. More importantly, Au@MC38 NPs demonstrated excellent in vivo MC38 tumor targeting, which enabled the reduction of Au@MC38 NPs dose for the similar level of tumor enrichment and radiosensitization effect in the MC38 tumor. To further demonstrate the in vivo generality of biosynthetic Au NPs for radiotherapy sensitization, Au@4T1 NPs were also synthesized for the treatment of orthotopic breast cancer. Au@4T1 not only exhibited an excellent in vivo radiotherapy sensitization in the 4T1 tumor model, but also inhibited the occurrence of lung metastases and greatly improved the survival of mice.

In a brief summary, metal‐based nanomaterials containing high‐Z elements such as Gd, Hf, Ta, Bi, W, and Au exhibit strong biological effects of radiotherapy sensitization leading to the enhanced deposition at tumor sites and increased radiosensitization of the tumor, and also improve the tumor hypoxia by directly generating O_2_ using H_2_O_2_ in TME or by increasing blood flow to tumor sites. Therefore, metal‐based nanomaterials can effectively improve the therapeutic effect of tumor treatment and reduce the damage to the normal tissues at the same time, which provides a promising idea to improve traditional tumor radiotherapy.

### 2.2 Metal‐enhanced catalytic therapy

TME refers to the surrounding microenvironment of tumor cells, including surrounding blood vessels, immune cells, fibroblasts, bone marrow‐derived inflammatory cells, various signaling molecules, and extracellular matrix (ECM), which are closely related to the occurrence, growth, and metastasis of the tumors.^[^ ^79^ ^]^ In solid tumors, a completely different microenvironment would be formed from normal tissue due to the rapid growth of tumor tissue. For example, tumor cells are only able to metabolize energy through anaerobic glycolysis due to the insufficient oxygen supply, which leads to the accumulation of lactic acid. At the same time, ion‐exchange proteins on tumor cell membranes also transport H^+^ inside cells to the outside of cells, avoiding self‐acidosis, reducing the pH of TME, and presenting an overall acidic environment. At the same time, given the unique metabolism of tumor cells, a high concentration of H_2_O_2_ accumulates at the tumor site effectively promoting the growth, proliferation, and metastasis of tumor cells.^[^ ^79^ ^]^ CDT, using TME condition to kill tumor cells, has attracted widespread attention as a new class of Fenton response‐based cancer therapy since first proposed in 2016.^[^ ^7^ ^]^

**FIGURE 2:**
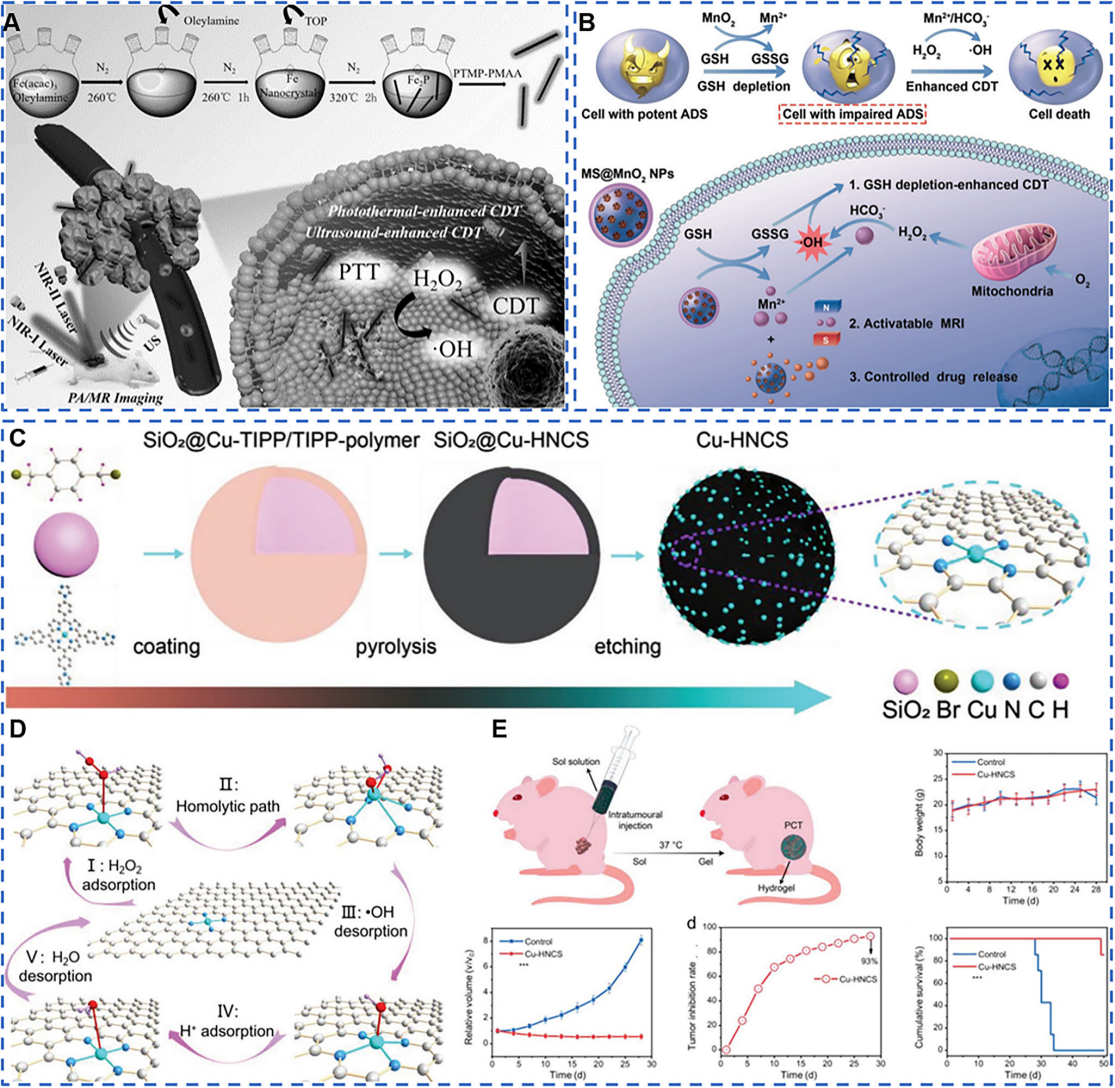
Metal‐based nanomaterials with catalytic biological effects for cancer therapy. (A) One‐dimensional FP NRs showed specific killing of tumor cells due to the presence of Fe^2+^. US irradiation accelerated the transformation of Fe^3+^ to Fe^2+^, and further enhanced the catalytic therapeutic effect. Reproduced with permission.^[^ ^29^ ^]^ Copyright 2019, Wiley‐VCH Verlag GmbH & Co. KGaA, Weinheim. (B) MS NPs (MS@MnO_2_ NPs) generated ·OH in response to the high concentration of GSH in the tumor, and the obtained Mn^2+^ enhanced the catalytic therapeutic effect and could be used in T1‐weighted magnetic resonance imaging. Reproduced with permission.^[^ ^35^ ^]^ Copyright 2018, Wiley‐VCH Verlag GmbH & Co. KGaA, Weinheim. (C–E) Single‐atom copper species performed superior anti‐tumor Fenton response than commercial Fe_3_O_4_. Reproduced with permission.^[^ ^27^ ^]^ Copyright 2020, Wiley‐VCH Verlag GmbH & Co. KGaA, Weinheim.

The Fenton reaction is a chain reaction between divalent iron ions (Fe^2+^) and H_2_O_2_ catalyzing the generation of ·OH with strong oxidative properties. The reaction needs to be carried out in high H_2_O_2_ concentration as well as the micro‐acidic environment, similar to the TME, which makes metal‐based nanomaterials with catalytic biological effects were able to be widely used in CDT for the purpose of killing tumors without causing normal tissue damage. Liu et al. obtained one‐dimensional (1D) ferrous phosphide nanorods (Fe_2_P) by high‐temperature synthesis and modified them with trithiol‐terminated poly(methacrylic acid) (PTMP‐PMAA) in the form of ligand exchange to obtain water‐soluble and biocompatible FP NRs (Figure 2A).^[^ ^29^ ^]^ FP NRs showed good stability in the physiological conditions and exhibited excellent catalytic effects, with significant killing effects on HeLa cells without causing significant damage to the normal L929 cells. However, as the Fe^3+^ obtained after the reaction of Fe^2+^ with H_2_O_2_ could not continue to participate in the subsequent catalytic therapy, the tumor‐killing efficiency of FP NRs was greatly reduced. To overcome this issue, Liu et al. also performed US irradiation of tumors in addition to i.v. injection of FP NRs to promote Fe^3+^ to Fe^2+^, thus achieving an enhancement of catalytic effect under US irradiation. More importantly, FP NRs exhibited a good T_2_‐weighted magnetic resonance imaging (MRI) effect due to the presence of iron, providing imaging navigation for FP NRs in tumor CDT treatment. Similarly, our group also prepared Fe‐doped TiO_2_ nanodots (Fe‐TiO_2_ NDs) using the high‐temperature organic‐solution method, in which the doped Fe^2+^ ions exhibited a good catalytic effect to potentiate the catalytic treatment.^[^ ^38^ ^]^

Unfortunately, the tumor‐killing effect of the Fe^2+^‐mediated catalytic effect is greatly reduced by the presence of high concentrations of GSH, with ·OH scavenging ability in the TME. In addition to Fe^2+^, scientists have identified more metal ions that could generate ·OH with H_2_O_2_, for example, Mn^2+^ and Cu^2+^. The reaction between other metals, except Fe^2+^, and H_2_O_2_ to generate ·OH is called Fenton‐like reactions. The reaction conditions of Fenton‐like reaction are similar to those of Fenton reaction, however, the other special conditions are required for the reaction with different elements. For example, for Mn^2+^, it requires the presence of HCO_3_^−^ in order to react with H_2_O_2_ in a Fenton‐like reaction. Therefore, it is a great urgency to develop metal‐based nanomaterials with biological catalytic effects, which simultaneously consume GSH in tumors while generating Fenton‐like reactions and achieve better tumor‐killing effects. Lin et al. formed manganese dioxide (MnO_2_) in situ on the surface of thiol‐functionalized mesoporous silica (MS) NPs by reacting thiol groups with an excess of permanganate, resulting in manganese dioxide coated MS NPs (MS@MnO_2_ NPs) (Figure 2B).^[^ ^35^ ^]^ MnO_2_ shell was able to react with GSH to generate GSSG and Mn^2+^ after entering tumor cells, and the obtained Mn^2+^ further reacted with H_2_O_2_ to generate ·OH under the physiological condition of HCO_3_^−^, which broke the antioxidant defense system (ADS) of tumor cells, enhancing the tumor Fenton‐like killing effect of MS@MnO_2_ NPs. Meanwhile, with the conversion of MnO_2_ to Mn^2+^, the efficiency of T_1_‐weighted MRI was significantly improved, providing T1 image navigation for the subsequent tumor treatment. More importantly, MnO_2_ decomposed only under high GSH conditions, allowing the anti‐tumor drugs loaded in mesoporous MS NPs to be only released at the tumor site, which served as a targeted tumor killing and reduced the damage of normal cells by anti‐tumor drugs. Thus, MS@MnO_2_ NPs showed an excellent killing effect on both in vitro and in vivo U87MG tumor. Iron‐doped vanadium disulfide nanosheets (Fe‐VS_2_ NSs) sharing the similar killing mechanism exhibited an enhanced catalytic therapeutic effect in tumor cells wherein Fe^2+^ possess good effect of catalyzing H_2_O_2_ to produce ·OH while the metastable metal V synergistically had an ability to rapidly deplete GSH. Iron element was able to perform an excellent T_1_‐weighted MR imaging contrast agent function, which provided MR image navigation for the catalytic treatment of Fe‐VS_2_ NSs.^[^ ^37^ ^]^

Due to the harsh reaction conditions of Fenton and Fenton‐like reactions, ordinary Fenton and Fenton‐like reagents are difficult to exert excellent anti‐tumor effects in the tumor CDT. Therefore, scientists are urgently seeking Fenton/Fenton‐like reagents with high catalytic efficiency to improve the efficiency of Fenton reaction and enhance the efficiency of tumor treatment. Single‐atom catalysts (SACs) were first proposed by Zhang et al. in 2011, and began to be studied due to their high catalytic efficiency.^[^ ^80^ ^]^ SACs are catalysts for the stabilization of isolated metal atoms on suitable carriers. The strong metal–support interaction between single‐atom and carrier interface in SACs effectively prevents single‐atom migration and aggregation, promotes a low coordination environment and charge transfer effect, and makes SACs have higher catalytic activity with a fully exposed active atom center. Meanwhile, the stability of SACs and the special properties of SAC activity depend on the selected substrate. SACs containing M‐Nx‐C sites obtained by anchoring single metal atoms on nitrogen‐containing carbon materials are similar to the M‐Nx sites of natural metalloenzymes, such as oxyhemoglobin, horseradish peroxidase (HRP), and cytochrome P450 enzymes. SACs have many similar characteristics to enzyme catalysts, especially in geometric and chemical structures, as well as electronic structures at the atomic level. More importantly, compared with natural enzymes and nano‐enzymes, SACs are widely used in the medical field due to their simple synthesis strategy, controllable active center and morphology, adjustable ligand environment, excellent catalytic activity, and low cost. Inspired by the multi‐copper oxidases (MCOs) consisting of multiple copper active sites coordinated by nitrogen and sulfur and based on the prepared silica nanospheres (SiO_2_ NSs) and Cu‐TIPP, Lu et al. generated SiO_2_@Cu‐TIPP/TIPP nanocomplex by quaternization reaction of SiO_2_ NSs, Cu‐TIPP, TIPP, and α, α'‐dibromo‐xylene. Finally, SiO_2_@Cu‐TIPP/TIPP polymers were pyrolyzed in a H_2_/Ar_2_ atmosphere and etched with NH_4_HF_2_ to obtain the Cu‐HNCS catalyst finally (Figure 2C).^[^ ^27^ ^]^ The Cu sites were uniformly distributed in the Cu‐HNCS catalyst and mainly presented as a Cu‐N_4_ structure. The Cu‐HNCS catalyst exhibited excellent Fenton‐like catalytic activity with 5000 times higher turnover frequency (TOF) than that of Fe atoms in the commercial Fe_3_O_4_. This excellent activity was verified by the Cu‐N_4_ active site (Figure 2D).^[^ ^27^ ^]^ More importantly, Cu‐HNCs catalyst could catalyze O_2_ to produce O_2_^−^ while showing a good Fenton‐like effect, leading to insufficient oxygen supply for tumor cells and resulting in enhanced killing effect of Cu‐HNCs catalyst on 4T1 tumor cells. To prevent the rapid leakage of Cu‐HNCS catalyst at the tumor site, Lu et al. injected the Cu‐HNCS catalyst into tumors by in situ gel formation to ensure the biocompatibility and therapeutic effect. Cu‐HNCS catalyst also exhibited an efficient Fenton‐like therapeutic effect in the 4T1 tumor model (Figure 2E).^[^ ^27^ ^]^

In short, the metal‐based nanomaterials containing reductive metals Fe^2+^, Cu^+^, Mo^5+^, V^5+^, and Mn^2+^ perform an ability to respond well to the micro‐acidic and high H_2_O_2_ concentration environment around tumors, exhibiting excellent Fenton/Fenton‐like biological effects to generate ·OH and achieving the purpose of killing tumor cells by using the characteristics of tumor cells. Since normal cells and tumor cells have completely different microenvironments, the damage to the normal cells caused by metal‐based materials with Fenton/Fenton‐like biological effects can be largely inhibited, which greatly reduce the side effects during the treatment and provide new ideas for the designing anti‐tumor drugs.

### 2.3 Metal‐enhanced ferroptosis

Both metal‐enhanced radiotherapy sensitization and metal‐enhanced catalytic treatment are able to directly and strongly kill tumor cells by producing ·OH with a strong killing effect on tumor cells, which may also cause tumor autophagy, improve the tolerance of tumor cells to stress, and greatly weaken the anti‐tumor effect. Therefore, we hope to find a tumor treatment that causes tumor cell death in an orderly and peaceful manner.^[^ ^81^ ^]^ Programmed cell death (PCD) was first proposed in 1951 and gradually became well accepted since then.^[^ ^82^ ^]^ PCD mainly includes apoptosis, cell necrosis, autophagy, and ferroptosis, among which ferroptosis has attracted extensive attention due to its ability to evade multiple drug resistance (MDR).^[^ ^83^ ^]^ Ferroptosis is a novel iron‐dependent, PCD, which mainly includes the following three mechanisms: (1) GSH‐based glutathione peroxidase 4 (GPX4) inactivation. (2) GPX4 inactivation: in addition to acting indirectly on GPX4‐activated GSH, GPX4 can also be eliminated directly.^[^ ^84^ ^]^ (3) Fe ion input and reduction: Fe ion is input into cells, and Fe ion is guaranteed to exist in large quantities in the form of divalent Fe, and divalent Fe are able to initiate liposome peroxidation through Fenton reaction.^[^ ^85^ ^]^ The characteristics of ferroptosis can be divided into the following two types. In terms of cell morphology, ferroptosis would lead to smaller mitochondria, increased membrane density, and decreased cristae. There was no obvious morphological change in the nucleus. In terms of cellular components, ferroptosis showed increased lipid peroxidation and ROS. Metal complexes are a kind of complexes formed by ligands and metal atoms or ions through coordination bonds. It is widely used in the preparation of metal‐based biomaterials because it is utilized to achieve specific purposes by replacing the desired metal according to the demand. Ferroptosis is an iron‐dependent mode of cell death, therefore, the synthesis of Fe‐based nanomaterials by introducing Fe through metal complexes has become a way to obtain ferroptosis inducers in tumor cells^[^ ^86^ ^]^; for example, 4‐substituted chloride [*N*,*N*’‐disalicylidene‐1,2‐phenylenediamine] iron (III) complexes, PtH@FeP obtained by HA‐cisplatin (PtH) cross‐linked complexes onto a Fe (III)‐polydopamine (FeP) core, Fe (III)‐shikonin supramolecular, PAMAM dendrimer‐iron (III) complex, and so on.^[^ ^43^ ^]^

**FIGURE 3:**
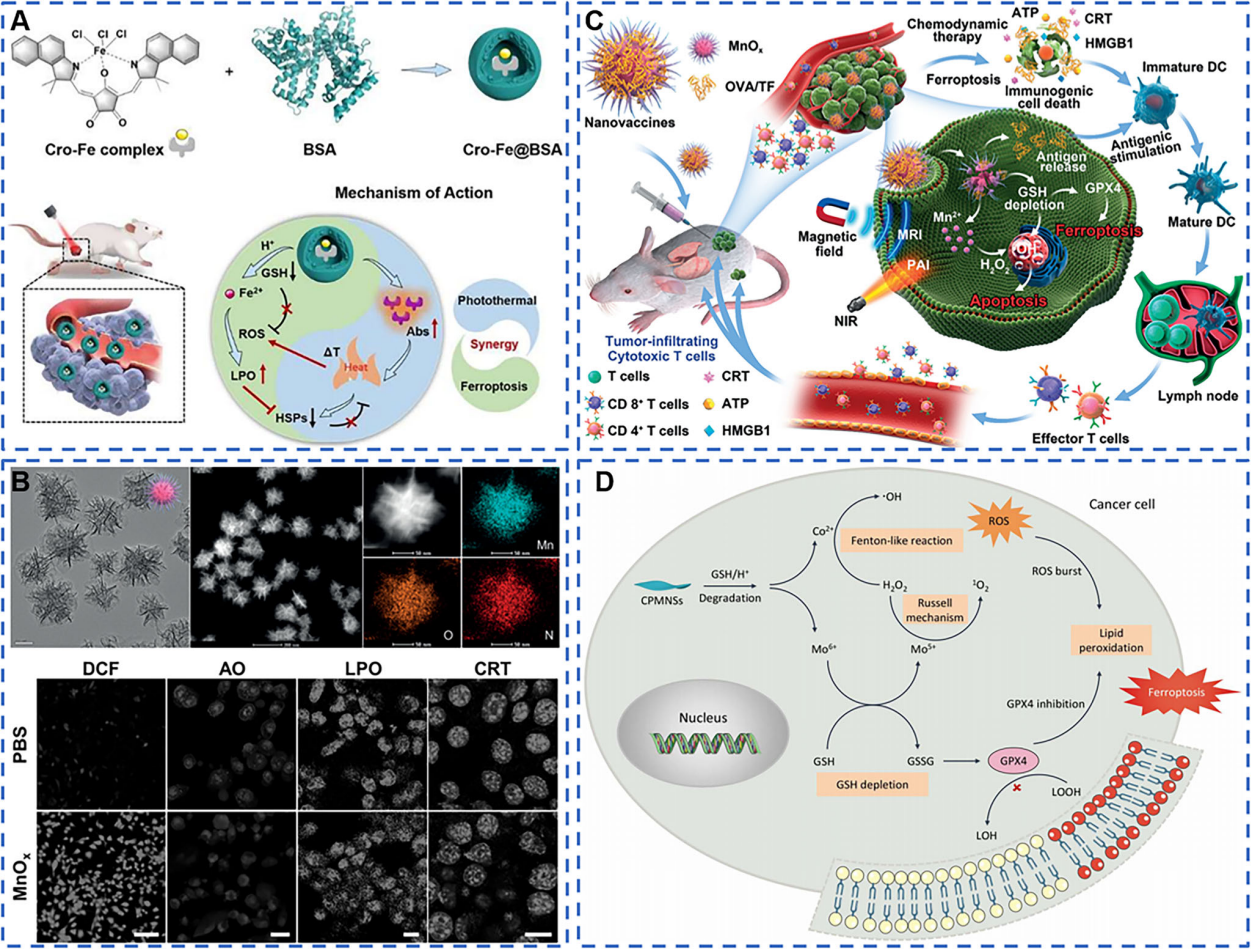
Metal‐based nanomaterials with ferroptosis biological effects for cancer therapy. (A) Acid tumor microenvironment (TME) accelerated the release of Fe^3+^ in Cro‐Fe@BSA, consumed glutathione (GSH), and decreased GSH‐based glutathione peroxidase 4 (GPX4) expression. The obtained Fe^2+^ further interacted with H_2_O_2_ to produce ROS, which accelerated lipid peroxidation and induced ferroptosis in tumor cells. Reproduced with permission.^[^ ^42^ ^]^ Copyright 2021, Wiley‐VCH GmbH. (B,C) MnO*_x_* Nanospikes were synthesized for cancer ferroptosis with Mn^2+^. Reproduced with permission.^[^ ^45^ ^]^ Copyright 2020, Wiley‐VCH Verlag GmbH & Co. KGaA, Weinheim. (D) CoMoO_4_‐phosphomolybdic nanosheets (CPMNS) were degraded gradually in acidic TME and under high GSH concentration to form Co^2+^ and Mo^6+^. Mo^6+^ consumed GSH and decreased the expression of GPX4 in cells. While Co^2+^ reacted with H_2_O_2_ to form ·OH. The simultaneous action of the two metal ions significantly increased the level of lipid peroxidation in tumor cells, and further caused ferroptosis in tumor cells. Reproduced with permission.^[^ ^41^ ^]^ Copyright 2021, American Association for the Advancement of Science.

Zeng et al. obtained Cro‐Fe complexes by coordination of croconaine (Cro) molecules and Fe^3+^, and then modified them with bovine albumin (BSA) to obtain Cro‐Fe@BSA with good stability and biocompatibility, which kept the valence of Fe ion in Cro‐Fe@BSA with +3 valence (Figure 3A).^[^ ^42^ ^]^ Meanwhile, the universal coordination ability of Cro and metal ions was also demonstrated. As the protonation of N in the acidic environment accelerated the charge leaving the domain, it would lead to a significant decrease in the binding affinity of Cro to Fe^3+^. Therefore, Cro‐Fe@BSA had an ability to react with high level concentration of GSH in the acidic TME of tumor cells, consume GSH, and degrade to obtain Fe^2+.^ All the functions together enabled the expression of GPX4 decrease, and the obtained Fe^2+^ further reacted with H_2_O_2_ in tumor to generate ROS increasing the level of lipid peroxide in tumor cells and decreasing the expression of heat shock protein 70 (HSP70), and finally induced 4T1 tumor cell ferroptosis. Cro‐Fe@BSA exhibited excellent ferroptosis anti‐tumor effects in a 4T1 tumor model. Furthermore, Fe released from Cro‐Fe@BSA was able to enhance T_1_‐weighted MR imaging, providing imaging navigation support for the subsequent induction of ferroptosis in the tumors.

According to the mechanism of ferroptosis, in addition to Fe, other metal‐based nanomaterials capable of causing GSH depletion and GPX4 inactivation can be utilized for tumor ferroptosis therapy. Since GSH is highly reductive, Mn, a multivariable valence metal with high valence and strong oxidative properties, is widely used in the preparation of ferroptosis inducers. MnO*_x_* nanospikes with large mesoporous structures and good antigen loading capacity were synthesized by a rapid hydration process (Figure 3B).^[^ ^45^ ^]^ It could be found that the MnO*_x_* NSs were biodegradable in the acidic environment of 4T1 tumor cells, where the high‐valent state of Mn would consume GSH, while the expression of GPX4 in tumor cells was significantly decreased. In contrast, the low valence state and the Mn obtained after reaction with GSH could interact with H_2_O_2_ to form ROS. Lacking the antioxidant capacity of GSH, the level of lipid peroxidation in tumor cells was significantly increased, causing damage to tumor cells and exhibiting an excellent ability to induce ferroptosis in tumor cells. More importantly, in order to enhance the in vivo anti‐tumor ability of MnO*_x_* NSs, Ding et al. also attached pattern antigens such as ovalbumin (OVA) or tumor cell fragment (TF) to MnO*_x_* NSs. MnO*_x_*‐OVA/TF NSs further induced tumor cells to release cellular debris, high mobility group protein 1 (HMGB1), and other signaling molecules after inducing ferroptosis in tumor cells, while releasing the pattern antigen OVA/TF to stimulate DCs maturation and further enhance the anti‐tumor effect. Meanwhile, due to the presence of Mn and the valence change of Mn, MnO*_x_* NSs also had the ability to enhance MR and photoacoustic (PA) imaging, which provided imaging support for the ferroptosis anti‐tumor therapy induced by MnO*_x_* NSs (Figure 3C).^[^ ^45^ ^]^

In many cases, metal‐based inorganic materials containing only one metal often could not play an excellent role in tumor therapy. To further enhance the ability of metal‐based nanomaterials to induce ferroptosis in tumor cells, nanomaterials containing multiple metals have also been designed and applied to induce ferroptosis in tumor treatment. CoMoO_4_‐phosphomolybdic acid nanosheets (CPMNS) were gradually degraded to generate Co^2+^ and Mo^6+^ under the conditions of tumor micro‐acidic environment and high GSH concentration (Figure 3D).^[^ ^41^ ^]^ Among them, Mo^6+^ had the ability to consume GSH to generate Mo^5+^ and reduce the expression of intracellular GPX4. Further, the obtained Mo^5+^ was able to react with a high concentration of H_2_O_2_ in the tumor to generate singlet oxygen (^1^O_2_), while Co^2+^ reacted with H_2_O_2_ in the presence of HCO_3_^‐^ to generate ·OH. The simultaneous actions of the two metal ions caused a significant increase in the level of lipid peroxidation in tumor cells, which further caused ferroptosis in tumor cells. Moreover, the redox of Mo and Co in CPMNS also accelerated the degradation of CPMNS and improved the tumor‐killing efficiency of CPMNS. More importantly, ferroptosis inhibitors such as Lip‐1 ferroptosis inhibitor and Desferrioxamine B mesylate (DFOM) iron chelator were also employed to confirm the inducement of CPMNS in tumor cell death through the ferroptosis pathway. Meanwhile, the tumor‐killing effect of CPMNS was also inhibited to some extent under the action of DFOM.

In summary, metal‐based nanomaterials contain oxidizing metals Fe^3+^ and Mo^6+^ or other metal elements with the function of inhibiting GPX4 enzymes, performing function as indirect GSH depletion by directly depleting GSH or inhibiting GPX4 enzymes after entering tumor cells, disrupting tumor cell redox homeostasis, and causing a significant increase in cellular lipid peroxidation levels, and finally induce ferroptosis in tumor cells. As a type of PCD, metal‐based nanomaterials with the biological effect of ferroptosis not only kill tumor cells, but also further improve the anti‐tumor effect by releasing damage‐associated molecular patterns (DAMPs), which in turn can serve to promote the ability of antigen‐presenting cells (APCs) to present antigens.

### 2.4 Metal‐enhanced pyroptosis

In addition to ferroptosis, another type of programmed tumor cell death, pyroptosis, has been noticed as its special morphological characteristics. Pyroptosis, known as inflammatory cell necrosis, are swollen and expanded under the light microscope, and have the protrusions of large bubbles from the plasma membrane. The pyroptosis cells form a large number of vesicles, namely pyroptosis corpuscles, before the rupture of the plasma membrane.^[^ ^87^ ^]^ After that, the pores would formed on the cell membrane, and the broken cell membrane releases the content. Pyroptosis mainly relies on the inflammasome to activate the part of proteins in the caspase family to cut gasdermin protein and activate gasdermin protein.^[^ ^88^ ^]^ The activated gasdermin protein is transferred to the membrane, forming holes, cell swelling, cytoplasmic outflow, and finally leading to cell membrane rupture and pyroptosis. In the classical approach, the adapter protein apoptosis‐associated speckle‐like protein (ASC) binds with the precursor of caspase‐1 to form a multi‐protein complex, which activates caspase‐1 and the activated caspase‐1 cleaves gasdermin D (GSDMD) and releases peptides in the active domain containing gasdermin C and gasdermin N, inducing cell membrane perforation rupture, releasing contents, and causing an inflammatory response. Meanwhile, the activated caspase‐1 cleaves the precursors of interleukin‐1β (IL‐1β) and interleukin‐18 (IL‐18) to form active IL‐1β and IL‐18, which are extracellularly released to recruit inflammatory cells and amplify the inflammatory response. Metal ions such as Fe^2+/3+^, Cu^2+^, and Mn^2+^ can break the redox balance in tumor cells, cause oxidative damage to tumor cells, induce the caspase family to cut gasdermin‐E (GSDME), and then form holes in the cell membrane, resulting in cytoplasmic outflow and pyroptosis.^[^ ^89^ ^]^

**FIGURE 4:**
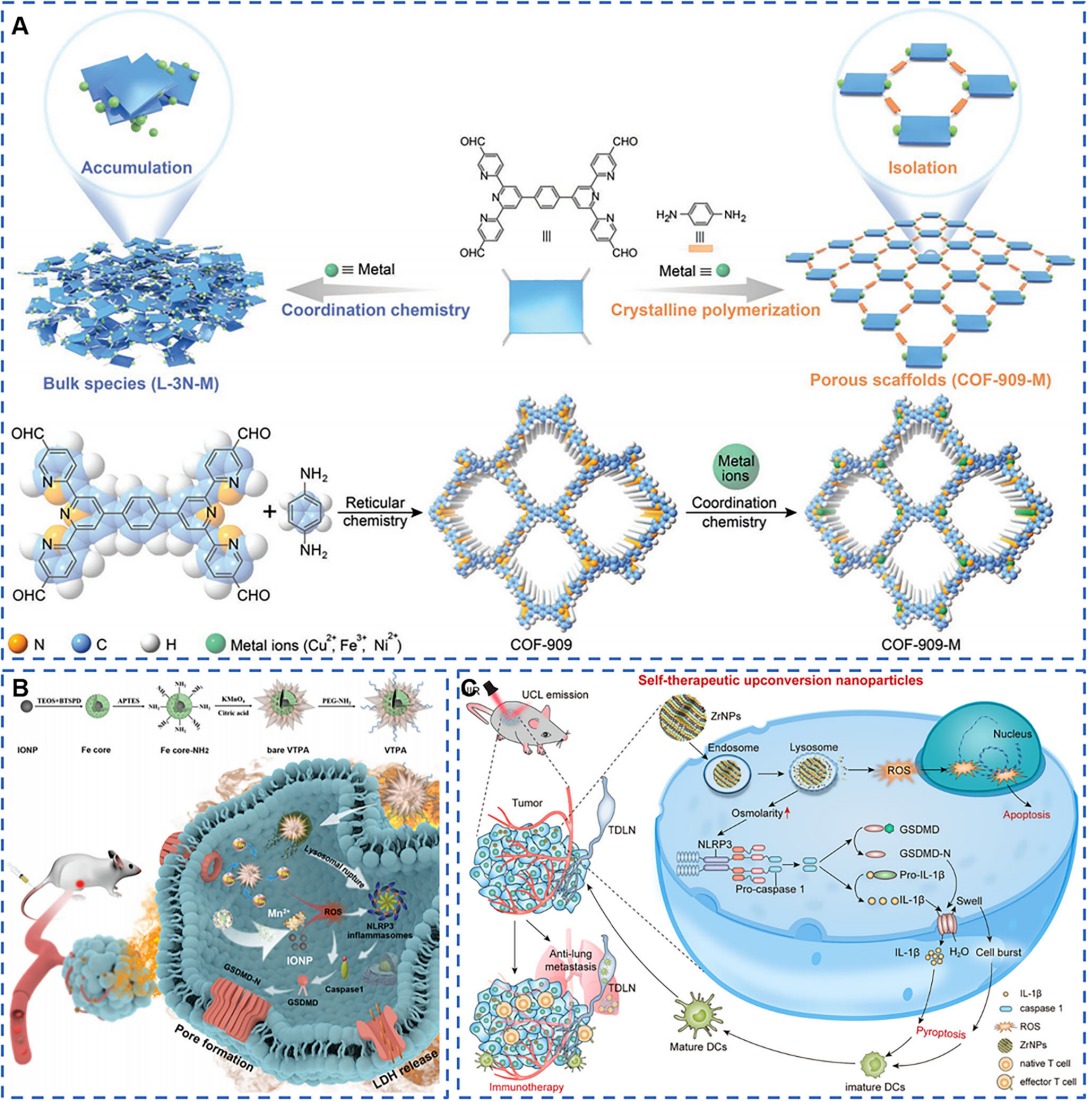
Metal‐based nanomaterials with pyroptosis biological effects for cancer therapy. (A) COF‐909‐Cu had better H_2_O_2_ interference ability, significantly increased the level of H_2_O_2_, consumed GSH, and showed the same activity of superoxide dismutase, GPx, and peroxidase (POD). Furthermore, COF‐909‐Cu‐induced gasdermin‐E (GSDME)‐dependent pyroptosis and significantly increased in cleaved caspase‐3 expression and cleaved and activated GSDME, leading to the formation of transmembrane pores, finally resulting in tumor cell pyroptosis and the release of large amounts of damage‐associated molecular patterns. Reproduced with permission.^[^ ^55^ ^]^ Copyright 2022, Wiley‐VCH GmbH. (B) Virus‐spike tumor‐activatable pyroptotic agent was synthesized for cancer pyroptosis with Mn and Fe. Reproduced with permission.^[^ ^56^ ^]^ Copyright 2022, Wiley‐VCH GmbH. (C) Spherical K_3_ZrF_7_:Yb/Er upconversion nanoparticles (ZrNPs) entered 4T1 tumor cells and released a large number of K^+^ and [ZrF_7_]^3−^ ions, resulting in intracellular osmotic pressure surge, ion overload, and enhanced intracellular oxidative stress. Oxidative stress further activated inflammatory vesicles and caspase‐1 proteins of nucleotide‐binding oligomeric domain‐like receptor protein 3 (NLRP3), leading to cleavage of GSDMD, and ultimately inducing cell pyroptosis. Reproduced with permission.^[^ ^52^ ^]^ Copyright 2021, American Chemical Society.

Covalent organic framework (COFs) materials are a class of porous crystalline organic materials discovered by Yaghi et al. in 2005.^[^ ^90^ ^]^ Organic building blocks are linked together by covalent bonds to form a porous skeleton with a periodic structure. These materials have excellent properties, with strong covalent forces between the skeletons, while COFs can combine with various metal elements through covalent interactions to perform the unique functions of metal ions. Zhang et al. constructed multiple metalloenzyme mimetic COFs such as COF‐909‐Cu, COF‐909‐Fe, and COF‐909‐Ni by post‐modification method (Figure 4A).^[^ ^55^ ^]^ Compared with COF‐909‐Fe and COF‐909‐Ni, the COF‐909‐Cu exhibited the optimum superoxide dismutase (SOD) activity and glutathione peroxidase properties. More importantly, COF‐909‐Cu exhibited better H_2_O_2_ interference ability, significantly increased intracellular H_2_O_2_ levels, consumed GSH, and also showed equally good SOD, GPx, and peroxidase (POD) activities. Furthermore, the tumor‐killing effect of COF‐909‐Cu was further promoted by 808 nm laser irradiation. COF‐909‐Cu performed an ability to induce cellular production of bubble‐like protrusions, which was one of the important morphological features of pyroptosis. Unlike the classical GSDMD‐dependent pyroptosis activated by caspase‐1, Zhang et al. found that COF‐909‐Cu‐induced GSDME‐dependent pyroptosis with a significant increase in cleaved caspase‐3 expression and cleaved and activated GSDME, leading to the formation of transmembrane pores, finally resulting in tumor cell pyroptosis and the release of large amounts of DAMPs.

Lysosomal rupture has been reported to accelerate the diffusion of ROS‐generating agents into the cytoplasm, and the ROS in turn oxidizes cysteine from GSDMD, enhancing the efficiency of caspase‐1 cleavage of cell pyroptosis. Thus, triggering both lysosomal rupture and ROS generation at the tumor site only is a promising approach to synergistically enhance the cell pyroptosis. In order to exploit the synergistic effects of multiple metals, scientists have started to design pyroptosis inducers containing multiple metals for application on the tumor pyroptosis therapy. Nadeem et al. decorated the surface of organosilica (OS) with a viral tip‐like structure of manganese dioxide (Mn) protrusions and modified it with PEG to obtain a virus‐spike tumor‐activatable pyroptotic agent (VTPA) with good stability and long blood circulation (Figure 4B).^[^ ^56^ ^]^ Moreover, the disulfide bond in VTPA performed a good GSH‐responsive function for TME‐responsive degradation, and the degradation of VTPA was a stepwise process from the outer layer to the inner layer. Furthermore, the Mn^2+^ and iron oxide nanoparticles (IONPs) released after VTPA degradation had the ability to catalyze the generation of ROS from H_2_O_2_ in the TME. VTPA was able to rupture the lysosome of tumor cells, and significantly increased the expression of trypsin B, NLRP3, caspase‐3, GSDMD‐N, and other inflammatory bodies and pyroptosis biomarkers, confirming the ability of VTPA to induce pyroptosis of tumor cells. More importantly, Mn shell and Fe core showed a good synergistic killing effect on tumor cells, but did not cause significant damage to the normal cells. VTPA also exhibited excellent tumor pyroptosis‐inducing function in the breast cancer in situ model. What is more, due to the presence of Mn and Fe, VTPA also exhibited doubly enhanced T_1_‐ and T_2_‐weighted MR imaging, providing image navigation for in vivo tumor treatment with VTPA.

Rare‐earth upconversion nanophosphors (UCNPs) are mainly obtained from inorganic substrates such as oxides, fluorides, and halogen oxides by doping with trivalent rare‐earth ions (Er^3+^, Eu^3+^, Yb^3+^, Tm^3+^, Ho^3+^, etc.). The unique frequency conversion capability makes UCNPs many advantages, such as high signal‐to‐noise ratio, long fluorescence lifetime, no light scintillation and photobleaching, low toxicity, narrow emission band and large penetration depth, and almost no damage to the biological tissues. At the same time, UCNPs are an excellent ion reservoir that can be loaded with multiple metal ions simultaneously to perform specific functions. Therefore, UCNPs are gradually applied to tumor pyroptosis therapy by disrupting cellular ionic homeostasis and altering cellular osmotic pressure. Ding et al. prepared spherical K_3_ZrF_7_:Yb/Er upconversion nanoparticles (ZrNPs) using thermal decomposition (Figure 4C).^[^ ^52^ ^]^ ZrNPs, as an ion pool with good biodegradable properties, had an ability to release a large amount of K^+^ and [ZrF_7_]^3−^ ions upon entering 4T1 tumor cells, causing a surge in intracellular osmotic pressure, occurring ion overload, inducing enhanced intracellular oxidative stress which further activated nucleotide‐binding oligomerization domain‐like receptor protein 3 (NLRP3) inflammatory vesicles and caspase‐1 protein, leading to the cleavage of GSDMD and finally inducing cell pyroptosis, while no significant killing effect was demonstrated in L929 normal cells. More importantly, the induction of pore formation, cell swelling, generation of large bubbles, and lysis by the gasdermin‐N structural domain caused by ZrNPs was clearly observed in tumor cells. Furthermore, the upconversion luminescence (UCL) properties of ZrNPs provide excellent UCL imaging navigation for inducing tumor pyroptosis and exhibiting excellent anti‐tumor ability.

In summary, metal‐based nanomaterials are able to induce tumor cell pyroptosis through caspase‐1‐dependent classical pathways or GSDME‐dependent non‐classical pathways by disrupting tumor cell ion homeostasis and redox homeostasis in tumor cells. They can be easily identified by their unique morphological features, such as the formation of a large number of vesicles (pyroptosis vesicles), the formation of pores in the cell membrane, the rupture of the cell membrane, and the flow of contents out of tumor cells.

### 2.5 Metal‐enhanced immunotherapy

In addition to utilizing TME response and inducing programmed tumor cell death, scientists focus on novel tumor therapies that stimulate systemic anti‐tumor immune responses in the matrix. Immunotherapy is a therapeutic method to artificially enhance or suppress the immune function of the body for the purpose of treating diseases.^[^ ^91^ ^]^ Tumor immunotherapy aims to activate the human immune system and relied on the autoimmune function to kill cancer cells and tumor tissue. The existing tumor immunotherapy can be divided into the following two categories: the utilization of drugs to relieve the immune suppression of the TME and to enhance the anti‐tumor immune response.^[^ ^92^ ^]^ To relieve immunosuppression in TME, immune checkpoint inhibitors such as anti‐PD‐1, anti‐PD‐L1, and anti‐CTLA‐4 are used to block immunosuppressive signaling pathways.^[^ ^93^ ^]^ The enhancement of anti‐tumor immune response can be achieved by activating the cGAS‐STING pathway and inducing immunogenic cell death (ICD) of tumor cells.^[^ ^94^ ^]^ Among them, Mn^2+^ and Zn^2+^ have been recognized as metal ions with activation function of cGAS‐STING pathway, while the overloading of Ca^2+^ performs an ability to induce ICD of tumor cells.^[^ ^95^ ^]^

ICD is a process in which tumor cells in a tumor undergo death by external stimulation and change from non‐immunogenic to immunogenic mediating the anti‐tumor immune response. When tumor cells undergo ICD, they generate a series of signaling molecules, known as DAMPs, mainly include calreticulin (CRT) exposed to the cell surface, HMGB1 secreted by tumor cells, ATP molecules released by cells, and heat shock proteins (HSP70 and HSP90).^[^ ^96^ ^]^ DAMPs released during ICD can bind to pattern‐recognition receptors (PRRs) on the surface of DCs, initiating a series of cytological responses that ultimately activate innate and adaptive immune responses. Taken together, immunotherapy that induces ICD in tumor cells is gradually recognized.

**FIGURE 5:**
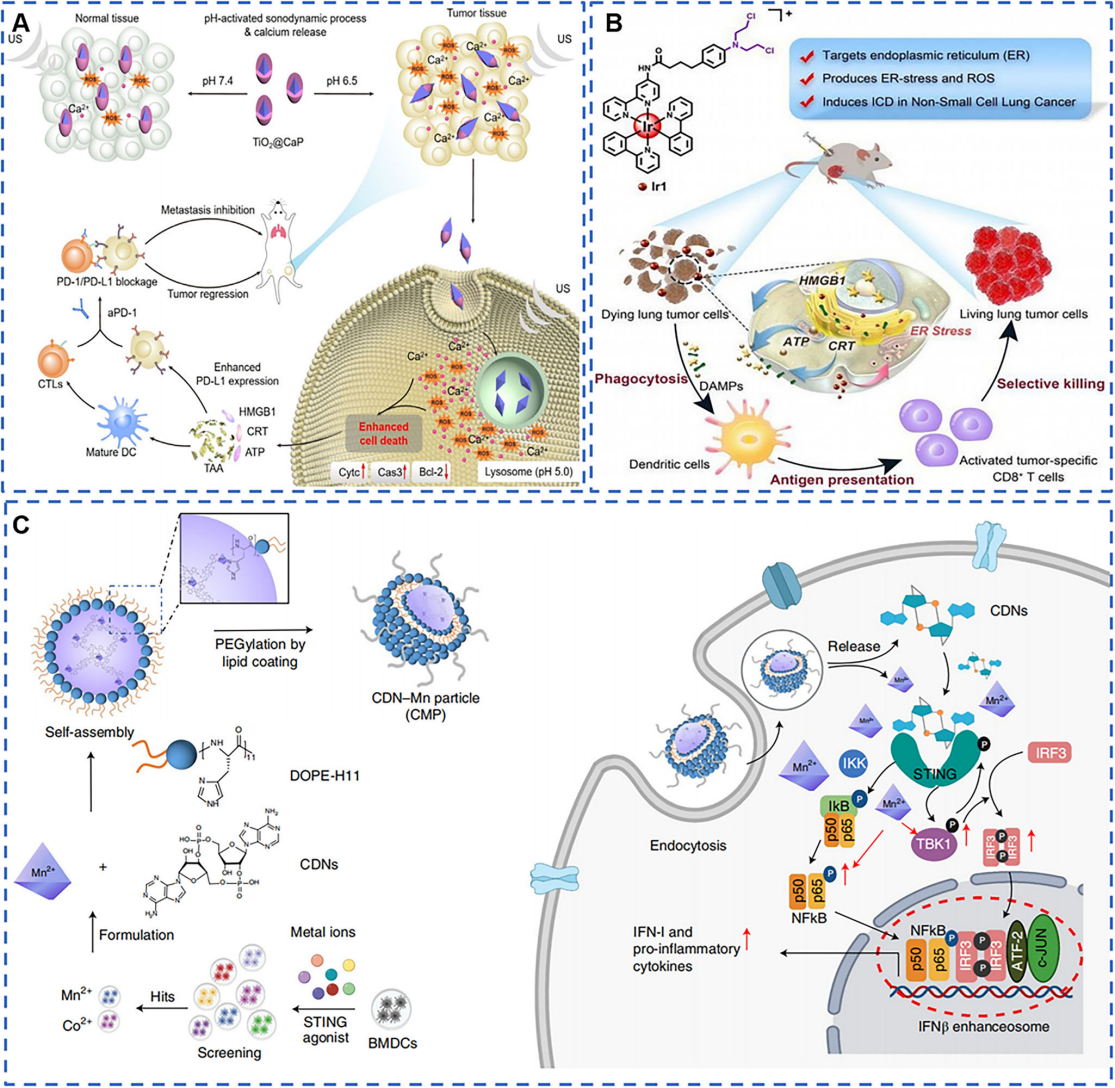
Metal‐based nanomaterials with immunotherapy biological effects for cancer therapy. (A) TiO_2_@CaP was able to enhance the anti‐tumor effect of immunotherapy by Ca^2+^. Reproduced with permission.^[^ ^64^ ^]^ Copyright 2021, Wiley‐VCH GmbH. (B) Iridium (III) complex Ir1 exhibited the ability to actively target tumor endoplasmic reticulum, causing severe damage to ER, increasing the phosphorylation level of eIF2α, promoting the release of Ca^2+^ in ER, and leading to mitochondrial Ca^2+^ overload. The expression of calreticulin on tumor cell surface was increased, and the extracellular migration of high mobility group protein 1 was promoted, showing immunogenic cell death. At the same time, the effect of anti‐tumor immunotherapy was enhanced by increasing the infiltration of CD8^+^ T cells in tumor sites. Reproduced with permission.^[^ ^66^ ^]^ Copyright 2020, Wiley‐VCH GmbH. (C) Because Mn^2+^ could amplify the activation of cGAS‐STING, CDN‐Mn particles (CMP) showed good anti‐tumor immunotherapy effect. Mn^2+^ amplified the activation of cGAS‐STING by increasing the phosphorylation levels of TBK1, P65, and IRF3. CMP enhanced the expression of interferon‐β, tumor necrosis factor‐α, and CXCL10 proinflammatory factors, and promoted the invasion of CD8^+^ T cells into tumors, showing a good therapeutic effect. Reproduced with permission.^[^ ^60^ ^]^ Copyright 2021, Springer Nature.

Calcium signaling is an important intracellular ion signal, and its intracellular concentration must be precisely regulated to achieve fidelity and accuracy of signaling. The endoplasmic reticulum (ER) is an important intracellular calcium reservoir, and maintaining a balance of Ca^2+^ concentrations in ER is essential for triggering accurate calcium signals and exercising normal cellular functions. A number of deleterious factors causes dysfunction of the calcium homeostasis system and disturbed calcium distribution, resulting in abnormally high intracellular calcium concentrations, namely, Ca^2+^ overloading. Ca^2+^ overloading has an ability to cause impaired oxidative phosphorylation processes in mitochondria, decreased mitochondrial membrane potential, decreased tissue ATP content, and activated intracytoplasmic phospholipases and proteases, which could promote irreversible cellular damage. The initiating calpain activation leads to the inward flow of extracellular Ca^2+^ via nifedipine‐sensitive calcium channels, followed by translocation to the cytosolic membrane, causing Cl^−^ inward flow and cell death, which is precisely ICD. Therefore, metal‐based nanomaterials that induce ICD in tumor cells by causing Ca^2+^ overload in tumor cells are widely used in tumor immunotherapy. Tan et al. prepared conical titanium dioxide nanoparticles (TiO_2_ NPs) by thermal decomposition and further coated them with CaP to obtain spherical TiO_2_@CaP NPs (Figure 5A).^[^ ^64^ ^]^ The CaP could degrade in the acidic TME, especially in tumor cell lysosomes, releasing large amounts of Ca^2+^ and reaching stability at about 6 h, while CaP coating exhibited good stability in the normal cellular environment. The released Ca^2+^ would specifically induce mitochondrial dysfunction in tumor cells, interfere with oxidative phosphorylation, and enhance ROS production in the cytoplasm, ending up damaging tumor cells. Furthermore, the TiO_2_ core was exposed after the degradation of CaP, which had a strong sonodynamic effect, making the sonodynamic therapy switch from the “off” state to the “on” state, further damaging the tumor cells. More importantly, Ca^2+^ released from CaP had the ability to cause the ectopic release of CRT to the cell surface and HMGB1 expression, promoting the uptake, processing, and presentation of tumor antigens by DCs and leading to the recruitment and infiltration of T cells. Moreover, TiO_2_@CaP NPs showed both in vitro and in vivo good killing effect on 4T1 tumor cells. Similarly, CaH_2_ nanoparticles obtained by liquid‐phase exfoliation method were able to react with H_2_O to form Ca^2+^, H_2_, and OH^−^ after entering tumors, of which H_2_ had an ability to be used for gas therapy to kill tumors, OH^−^ played a role in regulating the TME to potentiate cancer therapy, and the obtained of Ca^2+^ was able to cause Ca^2+^ overloading in tumor cells and potentiate tumor immunotherapy.

The existing ICD induction modalities are mainly divided into two types: one is to induce apoptosis by disconnecting with the ER, and to induce ICD‐related immunogenicity type I ICD by secondary ER stress effect. The other is type II ICD which induces ICD by directly altering ER homeostasis, triggering ER stress, and selectively targeting ER. Therefore, in addition to introducing Ca‐based nanomaterials to directly cause Ca^2+^ overloading in tumor cells, the endoplasmic reticulum is damaged by targeting the endoplasmic reticulum, resulting in oxidative stress and release of Ca^2+^ in the endoplasmic reticulum, and indirectly inducing Ca^2+^ overloading in tumor cells and inducing ICD have also been recognized. Wang et al. synthesized iridium (III) complexes Ir1 with bis(2‐chloroethyl)‐azidane and Ir2 without bis(2‐chloroethyl)‐azidane as reference compounds (Figure 5B).^[^ ^66^ ^]^ Ir1 showed an ability to actively target the tumor ER, causing severe damage to ER, increasing the phosphorylation level of eIF2α, promoting the release of Ca^2+^ in ER, leading to mitochondrial Ca^2+^ overload. It could further induce the opening of mitochondrial permeability filter pore (mPTP), destroy the dot transport chain, producing excessive ROS, and finally cause apoptosis. More importantly, Ir1 induced the rise of CRT expression on the tumor cell surface and promote the extracellular migration of HMGB1, demonstrating the ICD. Compared with Ir2, Ir1 showed the better selective killing of tumor cells and less damage to the normal cells. Ir1 demonstrated excellent in vivo anti‐LCC tumor activity and significantly prolonged the survival of mice. Moreover, Ir1 enhanced the effect of anti‐tumor immunotherapy by increasing the infiltration of CD8^+^ T cells at the tumor site. In addition to ICD inducers, Zn^2+^ also has the ability to induce CRT exposure on the cell membrane surface and release “eat‐me” signals to promote phagocytosis of dead tumor cells and maturation of APCs. Tian et al. synthesized ZnPI and modified it by PEG to obtain ZnPI‐PEG nanoparticles with good stability.^[^ ^97^ ^]^ ZnPI‐PEG NPs were able to weaken the coordination between Zn^2+^ and PI under acidic conditions due to the protonation process of nitrogen, in which Zn^2+^ could be released to induce strong CRT exposure on the surface of tumor cells, which had an ability to induce the similar levels to those of the classical ICD inducer OXA, effectively potentiating the subsequent metalloimmunology. In addition to the induction of ICD in tumor cells, tumor immunotherapy that directly targets immune cells was proposed in 2005. The cGAS‐STING pathway has been identified as an important pathway for activating immune cells and is widely used in tumor immunotherapy. When cGAS enzymes sense DNA that should not be present in the cytoplasm, they catalyze the production of small molecules of cyclic guanosine monophosphate (cGAMP), and dimerized STING binds to cGAMP, changing its conformation, recruiting TANK‐binding kinase 1 (TBK1) protein, phosphorylating, and activating interferon regulatory factor (IRF3), which induces type I interferon upon entry into the nucleus, and thus activating innate immunity.^[^ ^98^ ^]^ In addition, the cGAS‐STING pathway also reduces the expression of anti‐apoptotic protein BCL2 and upregulates the expression of pro‐apoptotic protein BAX in tumor cells, which then mediates the permeation of the mitochondrial outer membrane and activates caspase‐9‐derived caspase‐3, ultimately leading to apoptosis of cancer cells.^[^ ^99^ ^]^ In TME, the cGAS‐STING pathway in DCs plays an important role in promoting cross‐presentation and initiating tumor‐specific CD8^+^ T cells. Type I interferon produced in DC s plays the following functions: (1) To enhance the cross‐presentation of DCs. (2) By increasing the expression of CCR7 on DCs, the homing ability of lymph nodes was improved. (3) The homing effect of APC T cells can be transported by enhancing multiple Th1 cytokines. In general, the cGAS‐STING pathway plays an active role in the anti‐tumor immune response.^[^ ^100^ ^]^ By screening various metal cations, Sun et al. obtained Mn^2+^ and Co^2+^ with a good ability to amplify cGAS‐STING pathway activation, and assembled Mn^2+^ and STING agonist cyclic dinucleotide (CDN) by self‐loading, and then modified with DSPE‐PEG_5K_ to obtain biocompatible CDN‐Mn particles (CMP) (Figure 5C).^[^ ^60^ ^]^ The efficiency of CMP endocytosis was much higher than that of CDN. Meanwhile, due to the ability of Mn^2+^ to amplify cGAS‐STING activation, CMP showed better anti‐tumor immunity. Mn^2+^ amplified the activation of cGAS‐STING by increasing the phosphorylation levels of TBK1, P65, and IRF3. On the CT26 and B16F10 tumor models, both local and systemic, CMP enhanced the expression of interferon‐β (IFN‐β), tumor necrosis factor‐α (TNF‐α), and CXCchemokineligand‐10 (CXCL10) proinflammatory cytokines, and promoted tumor invasion of CD8^+^ T cells, thus showing a good therapeutic effect. Furthermore, CMP also performed superior therapeutic effect in the new tumor model squamous cell carcinoma model.

In addition, there are other ways to enhance tumor immunotherapy, such as activating T cells and natural killer (NK) cells. Chaigne‐Delalande et al. found that Mg^2+^ regulated the cytotoxic function of NK and CD8^+^ T cells via natural killer activating receptor natural killer group 2, member D (NKG2D).^[^ ^101^ ^]^ Meanwhile, Shi et al. reported that Ca^2+^ regulates T‐cell receptor activation by modulating the charge property of lipids.^[^ ^102^ ^]^ Although the activation ability of Ca^2+^ or Mg^2+^‐containing nanomaterials on NK and T cells has not been clearly reported in the literature, it also provides a new idea for the construction of metal‐based nanomaterials with enhanced tumor immunotherapeutic ability in the future.

In a brief summary, metal‐based nanomaterials containing Ca^2+^, Ir, and other metal elements that are able to directly or indirectly induce Ca^2+^ overloading in tumor cells exhibit a good biological effect of inducing ICD in tumor cells, which enhances anti‐tumor immunotherapy by targeting tumor cells. Meanwhile, metal‐based nanomaterials containing Mn^2+^, Zn^2+^, and other metal‐based materials with the ability to amplify the activation of the cCAS‐STING pathway exhibit a good ability to stimulate DCs maturation, which enhances the antigen‐presentation ability of DCs by directly targeting immune cells, while boosting the infiltration of CD8^+^ T cells at tumor sites for enhancing the anti‐tumor immune effect. Mg^2+^ and Ca^2+^ have the potential to regulate T cells and NK cells, providing a new idea for the application of metal‐based nanomaterials in tumor immunotherapy.

## 3 BIOSAFETY OF THE METAL‐BASED NANOMATERIALS

Metal‐based nanomaterials are a double‐edged sword. Although they largely exploit the properties of metals and enhance the efficiency of the tumor treatments, the risks of these materials regarding biosafety have always been a concern. Scientists have found the genotoxicity of nanomaterials that can pass the placental barrier from mother to fetus. Many metal‐based nanomaterials contain heavy metals that are difficult to be excreted from the body and are enriched in the kidney and liver, resulting in liver and kidney failure. Therefore, promising biosafety of metal‐based nanomaterials would be a prominent issue before their clinical translation.^[^ ^103^ ^]^

**FIGURE 6:**
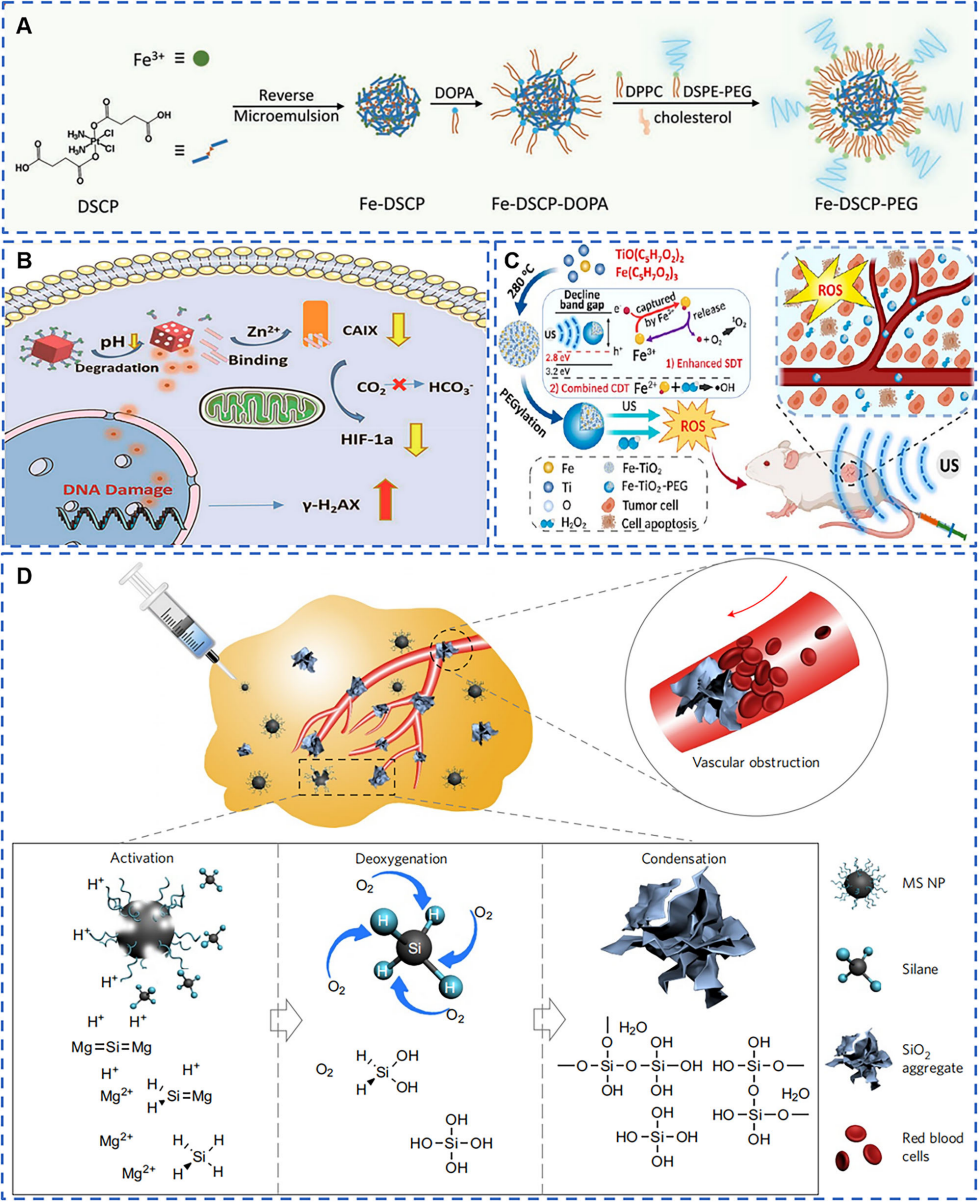
Biosafety of the metal‐based nanomaterials. (A) Fe‐DSCP‐PEG actively targeted tumors by modifying targeting peptide cRGD to improve biosafety. Reproduced with permission.^[^ ^104^ ^]^ Copyright 2020, Wiley‐VCH Verlag GmbH & Co. KGaA, Weinheim. (B) Zr‐MOF‐QU, which was able to degrade in the acidic environment of tumor, could effectively improve safety. Reproduced with permission.^[^ ^10^ ^]^ Copyright 2019, American Chemical Society. (C) Ultrasmall metabolizable Fe‐TiO_2_ could be discharged quickly after its catalytic therapeutic effect, greatly improving biocompatibility. Reproduced with permission.^[^ ^38^ ^]^ Copyright 2020, American Chemical Society. (D) Sodium chloride nanoparticles introduced the nutrient element Mg to improve biosafety. Reproduced with permission.^[^ ^105^ ^]^ Copyright 2017, Nature Publishing Group.

Nanomaterials, due to an EPR effect at tumor sites after i.v. injection, are difficult to avoid their massive enrichment in the liver and kidney. One effective method to relieve the issue is to design metal‐based nanomaterials with active tumor targeting. Liu et al. prepared novel biodegradable Fe‐DSCP nanomaterials based on Fe ion (III) and cisplatin prodrug by reverse microemulsion method, and modified it with targeted peptide cRGD to obtain Fe‐DSCP‐PEG‐cRGD with active targeting (Figure 6A).^[^ ^104^ ^]^ Fe‐DSCP‐PEG‐cRGD showed an excellent response to GSH and CDT. Fe‐DSCP‐PEG‐cRGD with i.v. injection demonstrated good tumor targeting.

In addition to targeting tumor sites with drugs or polymers, the preparation of nanomaterials using tumor cell membranes can also greatly improve the homing ability of nanomaterials. Zhu et al. designed a Fe_3_O_4_‐based nanoplatform coated with different cracked cancer cell membranes to obtain bionic nanomaterials with high recognition ability for homologous cells. Fe_3_O_4_@COS_7_, Fe_3_O_4_@UM‐SCC‐7, and Fe_3_O_4_@HeLa nanocomplex had demonstrated the homologous targeted tumor therapy, providing new ideas for the design of nanomaterials with active tumor targeting function^[^ ^106^ ^]^. To further improve the safety of metal‐based nanomaterials, erythrocyte membranes and other normal cell membranes have also been used to construct biomimetic nanomaterials. Xiong et al. added red blood cell (RBC) membrane on the basis of the tumor cell membrane to obtain a hybrid biomimetic coating (IRM), and further prepared indocyanine green (ICG)‐loaded magnetic nanoparticles (Fe_3_O_4_‐ICG@IRM) by IPM for synergistic photothermal‐immunotherapy.^[^ ^107^ ^]^ Fe_3_O_4_‐ICG@IRM preserved both RBC membrane and tumor cell membrane protein, which had highly specific self‐recognition of tumor cells and effectively activated anti‐tumor immunotherapy after photothermal therapy.

In addition to enhancing the targeting of metal‐based nanomaterials to tumors, the toxicity of metal‐based nanomaterials would be greatly reduced if the metal‐based nanomaterials are able to be quickly cleared from the matrix after the tumor killing. Therefore, the preparation of biodegradable and rapidly cleared metal‐based nanomaterials has become one of the most important methods to improve their biological safety. Song et al. prepared molybdenum oxide nanosheets by a one‐pot hydrothermal method and modified them with PEG to obtain biocompatible MoO*_x_*‐PEG nanosheets.^[^ ^108^ ^]^ MoO*_x_* had demonstrated good photothermal efficacy and was able to be used for PA imaging to provide image navigation for tumor therapy. More importantly, MoO*_x_* had an ability to be rapidly excreted from the body through kidney and stool within 7 days after i.v. injection. The Zr‐MOF‐QU synthesized by Ma et al. had the ability of acid‐responsive degradation, which degraded in the acidic TME. The mechanism of alleviating hypoxia‐induced resistance and improving apoptosis in sensitive tumor tissues could inhibit the expression of CAIX and improve the sensitivity of QU in radiotherapy. The acid‐responsive degradation ability of Zr‐MOF‐QU also greatly improved its biosafety (Figure 6B).^[^ ^10^ ^]^ Similarly, our group also prepared VS_2_ nanosheets by high‐temperature oil‐phase method, and then modified them with liposomes to reduce their degradation rate.^[^ ^109^ ^]^ The biodegradable VS_2_@lipid‐PEG NPs were able to be discharged through renal and fecal pathways within 30 days after i.v. injection. Ultrasmall, metabolizable metal‐based nanomaterials also effectively improve biosafety. Bai et al. synthesized Fe‐TiO_2_ nanodots with a size of about 3 nm by high‐temperature method, which not only played a highly effective catalytic therapeutic effect, but also could be rapidly metabolized out of the body due to its ultrasmall size, significantly improving its safety (Figure 6C).^[^ ^38^ ^]^

In order to reduce the introduction of heavy metals and trace elements in the process of cancer treatment, the synthesis of metal‐based nanomaterials using nutritional metal elements, such as magnesium and sodium, has gradually become an important approach to improving the biological safety of metal‐based nanomaterials.^[^ ^110^ ^]^ Zhang et al. used a special self‐propagating high‐temperature synthesis (SHS) method to prepare Mg_2_Si nanoparticles as oxygen scavengers (Figure 6D).^[^ ^105^ ^]^ Mg_2_Si nanoparticles were able to absorb oxygen and generate Mg^2+^, H_2_O, and SiO_2_ in the acidic tumor environment. As an excellent deoxidizer, Mg_2_Si nanoparticles performed good ability to block oxygen supply and had a good therapeutic effect on tumor starvation. More importantly, in addition to generating non‐toxic biological products Mg^2+^ and H_2_O, the SiO_2_ was also able to block tumor capillary and enhance the effect of tumor starvation therapy, providing a new idea for the application of metal‐based nanomaterials in starvation therapy. Sodium, a macronutrient in the matrix, maintains low intracellular sodium levels in mammalian cells and reduces extracellular sodium, such as immersing cells in a hypotonic solution, could lead to cytoskeleton destruction, cell cycle arrest, and cell lysis. Therefore, increasing the endocytosis of tumor cells to sodium has also become a method to kill tumor cells. Jiang et al. prepared sodium chloride nanoparticles (SCNPs) by microemulsion method and modified them with PEG to obtain PSCNPs.^[^ ^111^ ^]^ After entering tumor cells, PSCNPs were able to gradually degrade into Na^+^ and Cl^−^ and the large number of Na^+^ and Cl^−^ changed the osmotic pressure inside cells and overwhelmed the protective mechanism of cells, which not only induced tumor cell apoptosis, further caused high immunogenic necrosis of tumor cells to achieve enhanced cancer immunotherapy. The local administration of PSCNPs showed a good in vivo anti‐tumor effect without any toxic and side effects on the matrix.

## 4 CONCLUSION AND PROSPECTS

**FIGURE 7:**
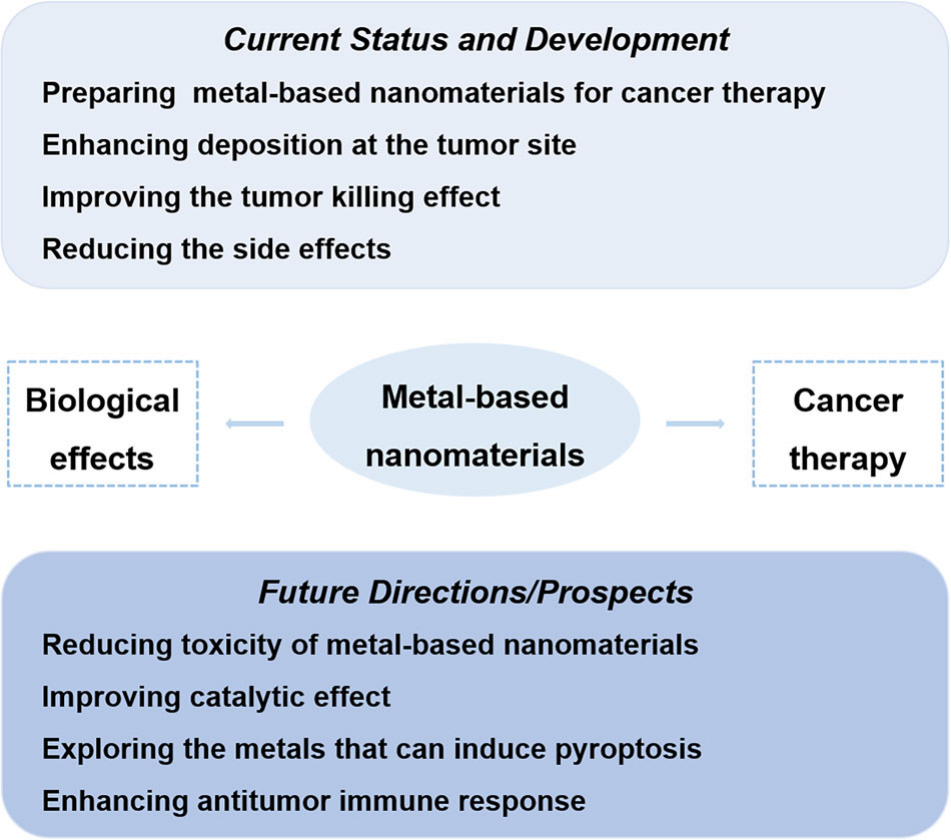
The current status and future prospects of metal‐based nanomaterials in cancer therapy.

Given to the unique physical and chemical properties of nanomaterials, some new tumor treatment methods relying on nanomaterials like radiotherapy sensitization, CDT, and immunotherapy have been rapidly developed. Among them, metal‐based nanomaterials are utilized in new tumor therapy by changing intracellular osmotic pressure, breaking cell redox balance or other homeostasis, or modulating T cell and macrophage phenotypes to enhance anti‐tumor immune responses. For example, Ca^2+^ can both modulate the T cell receptors activation and cause calcium overloading in tumor cells, thereby inducing tumor ICD. Fe^2+/3+^ is able to be used not only for tumor catalytic therapy but also to induce ferroptosis in tumor cells. Mn^2+^ and Zn^2+^ perform the ability to activate the STING pathway and enhance the anti‐tumor immune response. All of these make metal‐based nanomaterials crucial in novel tumor therapy. In this review, we summarized the recent progress of the anti‐tumor biological effects of metal‐based nanomaterials, including metal‐enhanced radiotherapy sensitization, catalytic therapy, ferroptosis, pyroptosis, and immunotherapy. Although the biological effects of metal‐based nanomaterials play a good anti‐tumor effect, it is worth noting that the relevant research is still in its infancy and many key issues remain to be solved (Figure 7).

The first is the safety of metal‐based nanomaterials, especially those with radiosensitizing effects. Due to the presence of high‐Z metal elements, although they perform better deposition in tumor sites, they will inevitably cause certain toxic side effects on the normal tissues. In addition, the metabolic pathways of heavy metal ions released after their degradation in vivo and their effects on the organism still need to be studied. Therefore, there is still a long way to apply metal‐based nanomaterials with radiosensitizing effects on clinical practice.

The second is the tumor‐killing efficiency of metal‐based nanomaterials, especially metal‐based nanomaterials with catalytic effect. Due to the strict reaction conditions of Fenton/Fenton‐like reaction, it is difficult to kill tumors by using the catalytic properties of metal‐based nanomaterials alone, therefore, it is common to add some external stimuli, such as US irradiation and laser, to enhance the efficiency of catalytic effect and thus improve the tumor‐killing effect. Alternatively, some other therapies may be combined to provide a combined or synergistic tumor‐killing effect. For example, Fe^2+^ participates in catalytic therapy, while Fe^3+^ can participate in the ferroptosis of tumor cells, so promoting the conversion between Fe^2+^ and Fe^3+^ is able to effectively enhance the anti‐tumor effect of Fe‐containing nanomaterials. Mn^2+^ plays an important role in both tumor catalytic therapy and immunotherapy, and the design of nanomaterials containing Mn^2+^ has great potential for enhancing tumor therapy. Therefore, the limit of metal‐based nanomaterials regarding strong catalytic activity still needs to be explored, and how to enhance the efficiency of tumor treatment by other external stimuli is still to be studied.

The third is the uncertainty of the mechanism of action of metal‐based nanomaterials, especially those with biological effects of pyroptosis. Although the pyroptosis pathway has been well studied, the specific metals or materials that could induce tumor cell scorch death have not been clearly investigated, so it is still very difficult to induce cell pyroptosis by introducing target metals.

The last is the non‐universality of metal‐based nanomaterials immunotherapy. Since metal‐based nanomaterials with immunological effects are more likely to enhance anti‐tumor immunotherapy effects by directly targeting immune cells, the toxic side effects of such metal‐based nanomaterials on immune cells have not been studied very clearly. More importantly, due to the individual differences, the biological effects of metal‐based nanomaterials are not yet completely universal, which greatly reduces the effectiveness of anti‐tumor therapy. Therefore, there is a need to combine therapeutic modalities more, which can effectively kill tumor cells while activating the body's anti‐tumor immune response, so as to achieve the purpose of enhancing the tumor‐killing effect. Therefore, the design and development of metal‐based nanomaterials with both tumor‐killing effect and enhanced immune response are still to be studied in depth.

In summary, studies on these metal‐based nanomaterials with various anti‐tumor biological effects have shown that these materials have promising applications. Thus metal‐based nanomaterials are expected to be applied on more aspects of the medical field. These explorations still require great efforts from more researchers. Although there is still a long way to go for the clinical application of metal‐based nanomaterials, they are certainly providing the directions and ideas for the development of novel therapeutic modalities for tumors.

## CONFLICT OF INTEREST STATEMENT

The authors declare no conflicts of interest.
